# Thalamic Control of Human Attention Driven by Memory and Learning

**DOI:** 10.1016/j.cub.2014.03.024

**Published:** 2014-05-05

**Authors:** José de Bourbon-Teles, Paul Bentley, Saori Koshino, Kushal Shah, Agneish Dutta, Paresh Malhotra, Tobias Egner, Masud Husain, David Soto

**Affiliations:** 1 Division of Brain Sciences, Department of Medicine, Imperial College London, Charing Cross Campus, St. Dunstan’s Road, London W6 8RP, UK; 2 Center for Cognitive Neuroscience and Department of Psychology & Neuroscience, Duke University, Levine Science Research Building, Box 90999, 450 Research Drive, Durham, NC 27708, USA; 3 Department of Experimental Psychology, University of Oxford, South Parks Road, Oxford OX1 3UD, UK

## Abstract

The role of the thalamus in high-level cognition—attention, working memory (WM), rule-based learning, and decision making—remains poorly understood, especially in comparison to that of cortical frontoparietal networks [1–3]. Studies of visual thalamus have revealed important roles for pulvinar and lateral geniculate nucleus in visuospatial perception and attention [4–10] and for mediodorsal thalamus in oculomotor control [11]. Ventrolateral thalamus contains subdivisions devoted to action control as part of a circuit involving the basal ganglia [12, 13] and motor, premotor, and prefrontal cortices [14], whereas anterior thalamus forms a memory network in connection with the hippocampus [15]. This connectivity profile suggests that ventrolateral and anterior thalamus may represent a nexus between mnemonic and control functions, such as action or attentional selection. Here, we characterize the role of thalamus in the interplay between memory and visual attention. We show that ventrolateral lesions impair the influence of WM representations on attentional deployment. A subsequent fMRI study in healthy volunteers demonstrates involvement of ventrolateral and, notably, anterior thalamus in biasing attention through WM contents. To further characterize the memory types used by the thalamus to bias attention, we performed a second fMRI study that involved learning of stimulus-stimulus associations and their retrieval from long-term memory to optimize attention in search. Responses in ventrolateral and anterior thalamic nuclei tracked learning of the predictiveness of these abstract associations and their use in directing attention. These findings demonstrate a key role for human thalamus in higher-level cognition, notably, in mnemonic biasing of attention.

## Highlights

• Pivotal role of human thalamus in linking memory and attention in vision
• Thalamus lesions disrupted attention biases by working memory contents
• Ventrolateral (VL) thalamic lesions reversed the normal direction of these biases
• Flexible scope of memory types biasing attention through VL and anterior thalamus

## Teaser

de Bourbon-Teles et al. present causal evidence from focal brain lesions and fMRI evidence from healthy participants showing that ventrolateral and anterior thalamic nuclei play a pivotal role in linking mnemonic and attention control functions in human vision.

## Results

We assessed a group of thalamic patients in a task probing the interaction between working memory (WM) contents and visual attention [16, 17], and then we used fMRI in healthy volunteers to test predictions derived from the lesion data. The study was approved by the West London Research Ethics committee.

### Patient Study

**Figure 1:**
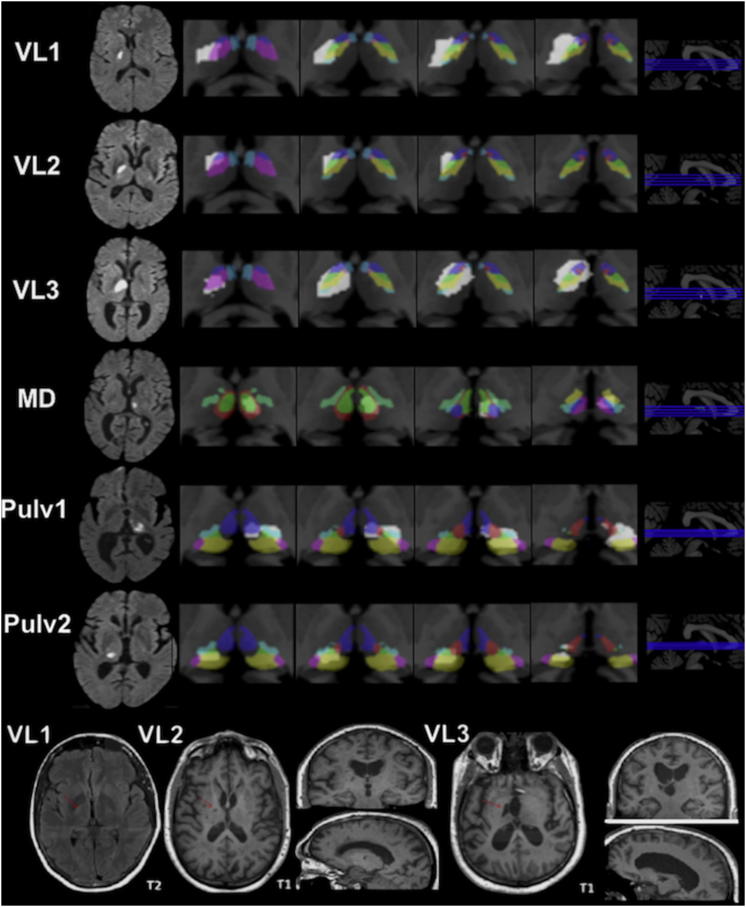
Lesion Maps of Thalamic Patients For the sake of simplicity, patients (n = 6) are labeled by the name of the thalamic lesion site showing overlap across them. Diffusion weighted imaging (DWI) scans (left column) illustrate the thalamic lesion site in the acute stage. Individual lesions were mapped onto a 3D, high-resolution, histology-based atlas of thalamic areas [18]. Axial slices to the right depict the thalamic nuclei in different colors, and the lesion area is highlighted in white. Table S1 depicts the percentage of damage of each thalamic nucleus and connectivity information from a diffusion tensor imaging atlas of probabilistic connections between thalamic nuclei and cortical regions [14]. The critical VL lesions included VLa, VApc, VLpv, and VLpd, which are densely connected with PFC [14]. The bottom row depicts high-resolution structural MRI of the VL patients acquired during the chronic stage following stroke. For patient VL1, we present the T2 brain scan rather than the T1 brain scan. Note that patient VL2 also had a small lesion in the left pallidum. Details: VL patients’ lesions involved VLa (green), VLpv (yellow), VLpd (violet), VApc (blue), VPla (light cyan), and VAmc (red). Note that only the VL3 lesion involved part of the anterior nuclei (AD and AV; dark cyan). The lesions in our MD patient mainly involved MDpc (green), CL (red), CM (blue), Pf (violet), VM (yellow), VPM (cyan), and VPLp (light green). The pulvinar patients’ lesions involved PuA (green), PuL (violet), PuM (yellow), CM (red), MDpc (blue), and VPLp (cyan). The following abbreviations are used: VAmc, ventral anterior magnocellular; VApc, ventral anterior parvocellular; VLa, VL anterior; VLpd, VL posterior dorsal; VLpv, VL posterior ventral; VPla, ventral posterolateral anterior; MDpc, mediodorsal parvocellular; Pf, parafascicular; CL, central lateral; CM, central medial; VM, ventral medial; VPM, ventral posteromedial; PuM, medial pulvinar; PuL, lateral pulvinar; PuA, anterior pulvinar.

**Figure 2:**
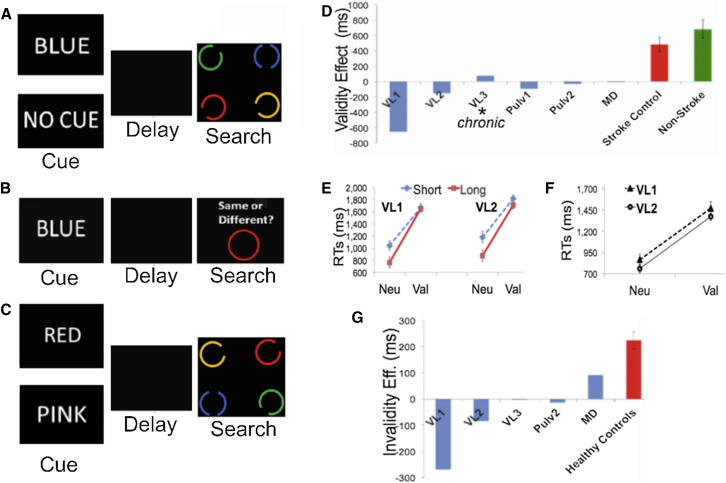
Patient Study: Trial Examples and Behavioral Data (A) Memory-guided search task in experiments 1, 2, and 3. Participants were given either the name of a color cue, which always matched the search target (100% valid), or a neutral cue (“no cue”). This was followed by a search task that included finding the circle containing two gaps in the vertical plane and reporting whether it was located to the left or to the right of the central fixation. Participants responded via a button press. (B) Delayed recognition task. In experiments 1 and 2, the ability to maintain the memory cue was assessed in a separate recognition task. Participants were required to remember the color word, and, following a 2 s delay, they were required to respond whether the colored circle matched or did not match the verbal cue. Experiments 3 and 4 incorporated a memory test after the search to ensure that the cue was held in memory across the delay. (C) Invalid cueing in experiment 4. If the color word matched an item presented in the search display, this would never be the item surrounding the target (100% invalid trials). (D) Experiment 1: Cue-validity effects on search (median neutral RT − median valid RT) for the thalamic patients and for the control groups (error bars show SEM of the cue-validity effect). The size of the cue-validity effects was reduced in the thalamic group relative to age-matched patients with lesions outside the thalamus (n = 18; t(22) = −3.5; p = 0.002; independent sample two-tailed t test) and in the nonstroke group of age-matched controls (n = 22; t(26) = −3.4; p = 0.002). Individual median RT data and SEM across the different validity conditions are presented in Table S2. (E) Experiment 2: Search RTs as a function of cue-validity (Neu, neutral; Val, valid) and cue-search delay in patients VL1 and VL2. Note that performance of the patients in the short delay condition was compared with the performance of control groups from experiment 1, which had been tested with the same delay. Cueing effects differed significantly between the patients with lesions outside the thalamus and the two VL patients, and the same held for the comparison with the nonstroke group (t > 11, p < 0.0001). (F) Experiment 3: Search RTs as a function of cue validity in the version of the task that incorporated a memory test after the search. VL patients showed reversed validity effects relative to the nonstroke controls and the stroke control group (all one sample t > 10, p < 0.0001). (G) Experiment 4: Cue-invalidity effects (invalid RT − neutral RT) for the thalamic patients and the healthy controls. Importantly, we only analyzed search trials with correct recognition memory responses. Healthy controls (n = 11) displayed slower search in invalid trials relative to neutral trials (F(2,10) = 50.47; p = 0.0001). Invalidity effects were significantly lower in the thalamic patients than in the healthy controls (t(26) = −3.38; p = 0.002). This pattern of results held when the data were transformed to account for overall individual search latencies (Supplemental Experimental Procedures).

Lesion maps appear in Figure 1. Figures 2A–2C depict the experimental paradigm (see also Supplemental Experimental Procedures available online). Thalamic patients’ performances were compared to 18 stroke controls (see Figure S1 for lesions) and 22 subjects without stroke who were admitted to the hospital for neurological evaluation.

#### Experiment 1

We tested whether thalamic lesions disrupt WM cueing effects on search. Analyses of cue validity effects (neutral reaction time [RT] – valid RT) showed that control groups used the cues strategically to boost search. Thalamic patients consistently failed to do so, showing reduced validity effects relative to controls (Figure 2D; for statistics, see figure legend). Results held when cueing effects were transformed in order to account for interindividual variation in reaction time (RT) (i.e., (neutral RT − valid RT)/(neutral RT + valid RT); see Supplemental Experimental Procedures). No effects of target visual field (i.e., contralesional versus ipsilesional) were found here or in subsequent experiments.

Intriguingly, two ventrolateral (VL) patients displayed a “reversed” validity effect, with slower performance in valid versus neutral trials. Patient VL3 showed a validity effect in the “normal” direction; however, its size was reduced relative to controls. We confirmed that validity effects in the control groups were higher than they were in each individual thalamic patient (all one sample t > 4, p < 0.0001; see also Figure S2A for individual data).

Notably, patients VL1 and VL2 were tested in the acute stroke phase in the present experiment and subsequently also in the chronic phase (up to 1 year later; see experiments 2–4). Testing in both acute and chronic phases mitigated the possibility that our findings relate to functional or maladaptive plasticity. Patient VL3, however, took part in the first experiment 5 years poststroke. Hence, due to this longer recovery period, it is likely that compensatory mechanisms may have operated to regain some of the functional loss in this patient.

Importantly, delayed recognition performance was high in the thalamic group (94.6% correct) and in the two control groups (95.4% and 94.4% correct, respectively) with no difference among control groups (for all: p > 0.8). The inability of thalamic patients to use the cue to guide search could thus not be explained by inability to retain it.

#### Experiment 2

Given the striking reversed validity effect in patients VL1 and VL2, we sought to replicate this in experiment 2. Patients were reexamined 6 months and 10 months after experiment 1, respectively (Figure 1; bottom row confirms the chronic stage of VL lesions). We also varied the delay (2 s versus 6 s) between cue and search displays to assess whether the absence of a cueing effect in experiment 1 could be improved by allowing the patients to have more time to use the cue.

Again, VL patients showed a reversed validity effect, which was not modulated by the delay between cue and search (see Figure 2E). Notably, memory of the cue was intact in the two VL patients (100% correct).

#### Experiment 3

In experiments 1 and 2, memory was assessed separately from the search task. It is possible that thalamic patients did not strongly commit the cue to WM despite it being search relevant and despite encouragement to use it strategically. Note, however, that this account would have predicted mere attenuation or absence of the WM bias rather than the reversed validity effect displayed by VL patients. Here, we included a memory test following the response to the search display in order to ensure that cues were in WM throughout the trials. Although delayed recognition memory was at ceiling (VL1 = 100% correct; VL2 = 97% correct), the same reversed validity effect was found (Figure 2F; see also Supplemental Experimental Procedures for a replication experiment).

This seemingly paradoxical effect is consonant with the view that thalamic insult triggers inhibition of any perceptual input that matches WM contents. We tested a crucial implication of this hypothesis in experiment 4.

#### Experiment 4

In experiments 1–3, WM contents were search relevant. However, recent research indicates that WM can automatically bias attention even when WM contents are irrelevant and detrimental in search [16, 17]: search is impaired when the WM content reappears as a search distracter as opposed to when it is absent [16]. This effect is contingent on participants holding the cue in WM because no attention bias is apparent when cues are merely attended (even in neurological populations [19, 20]; see [16, 17] for reviews).

To test the hypothesis that VL lesions result in the inhibition of memory-matching items, experiment 4 employed cues that were consistently invalid in search (Figure 2G). If the hypothesis was correct, then the patients would display better search performance than healthy controls in this invalid cueing protocol because the patients’ attention would be repelled by rather than be attracted to the memory-matching distracters. We tested VL patients and healthy controls, along with patients Pulv2 and MD, who acted as a refined control for testing whether the VL patients specifically show a reversed invalidity effect.

We analyzed the cue-invalidity effects (invalid RT − neutral RT). Whereas healthy controls (n = 11) displayed attentional capture by irrelevant WM contents (i.e., slower search on invalid versus neutral trials), thalamic patients did not exhibit attentional capture (see Figures 2G and S2B for individual data).

Most importantly, the two VL patients now displayed a reversed invalidity effect and responded faster in invalid trials compared to neutral trials (see Figure 2G). Notably, memory performance in these patients was high (VL1: 98% correct; VL2: 97% correct; VL3: 97.3% correct; Pulv2: 90% correct; MD: 85% correct).

Experiments 3 and 4 were conducted several months after stroke. We reiterate that patients VL1 and VL2 showed a similar reversed validity effect in the chronic and more acute stages, suggesting that the effect is unlikely to be the result of functional or maladaptive plasticity. Future research, however, ought to assess whether and how focal thalamic damage can trigger maladaptive functional reorganization changes in brain networks, which may further account for the impaired WM biasing of attention reported here.

### fMRI Studies

#### Experiment 1: Anterior Thalamus Is Involved in WM Biasing

Given the limited sample size of our rare thalamic patients, we further probed the role of the VL and anterior thalamus in WM biasing of attention by using fMRI in healthy participants. Note that only one of the VL patients’ lesions (i.e., VL3) comprised the more anterior thalamic nuclei (i.e., anterior dorsal [AD] and anterior ventral [AV]; Figure 1 and Table S1), described as part of a memory network including the hippocampus [15]. We propose that anterior nuclei, along with VL thalamus’s contribution to action and oculomotor control [12, 13, 21, 22], may represent a nexus between mnemonic and attention control functions.

**Figure 3:**
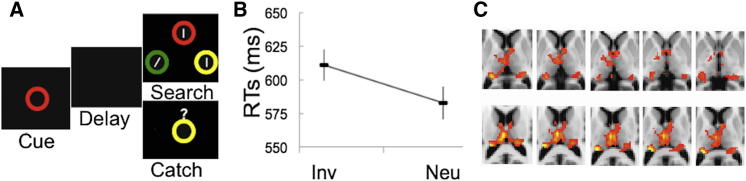
fMRI Experiment 1 (A) Task: A visual cue to be held in memory is presented. Following a delay, the search display appears. The task is to discriminate the orientation of the tilted bar (i.e., \ or /). An example of invalid trial is presented. Invalid trials were compared to a neutral baseline in which the cue was absent from search. In memory catch trials (20%), the search array was replaced by a recognition memory test. (B) Search RTs across invalid and neutral trials (error bars show SEM). Search was impaired by the presence of an invalid WM distracter relative to the neutral baseline (t(38) = 7.08, p < 0.00001, two-tailed t test; Figure 3B), in keeping with an automatic bias of attention by WM contents. Memory accuracy was high (mean = 93% correct). Search accuracy was high (mean = 93.75% correct) and did not differ across the neutral and invalid trials (t(38) = −0.842; p > 0.4). (C) Thalamic responses were enhanced by the reappearance of the cue relative to the neutral baseline.

We tested this hypothesis further in an fMRI study of healthy volunteers (n = 39) by using a paradigm that assessed the automatic biases of attention through irrelevant WM content [23, 24] that was similar to experiment 4 except that (1) here, the cues were visual, (2) search displays were brief (0.1 s) to prevent saccades, and (3) the search target was a tilted line (/ or \) among vertical distracters (see Figure 3A and Supplemental Experimental Procedures).

Behavioral results were consistent with automatic WM biasing of search (Figure 3B). Given our a priori interest in the thalamus, neuroimaging analyses used masks of the right and left thalami. Relative to the neutral baseline, there were increased responses to the reappearance of a WM distracter in bilateral pulvinar thalamus (medial pulvinar [PuM], lateral pulvinar [PuL], and inferior pulvinar [PuI]), mediodorsal thalamus (mediodorsal parvocellular [MDpc]), and, more critically, (1) VL regions overlapping with the patients’ lesion sites (ventrolateral anterior [VLa], ventrolateral posterior dorsal [VLpd], and ventral anterior parvocellular [VApc]) and (2) the more anterior thalamus bilaterally (including AV, anterior medial [AM], and AD) (see Figure 3C). These results survived correction for multiple comparisons within the thalamic regions of interest (ROIs) and across the whole brain. We also found activations in frontoparietal regions (Figure S3) and in the bilateral hippocampus (Montreal Neurological Institute [MNI]: 24, −20, −12 and −24, −26, −14). Given our a priori hypothesis concerning the thalamus, these activations are not discussed in great depth because they only provide correlational evidence, which, unlike our lesion evidence, precludes the formulation of causal inferences. We note that parietal and hippocampal responses have been recently associated with the strategic cognitive control over WM biases [25], and frontoparietal responses are classically involved in attention control [1].

Importantly, prior work has demonstrated that reappearance of WM contents is associated with increased neural responses relative to a nonrepetition baseline, whereas priming is associated with neural repetition suppression [26, 27]. Notably, we found no evidence for reduced responses to the reappearance of the memory cue in search. This is consistent with memory biases in this paradigm being contingent on WM [16, 17].

#### Experiment 2: Role of VL-Anterior Thalamus in Attention Biases Driven by Learning and Retrieval from Long-Term Memory

So far, the findings indicate a thalamic role in WM-based attentional control. However, it remains possible that the thalamus’s role in attention may incorporate additional mnemonic processes, such as when information from long-term memory is brought “online” to guide behavior.

**Figure 4:**
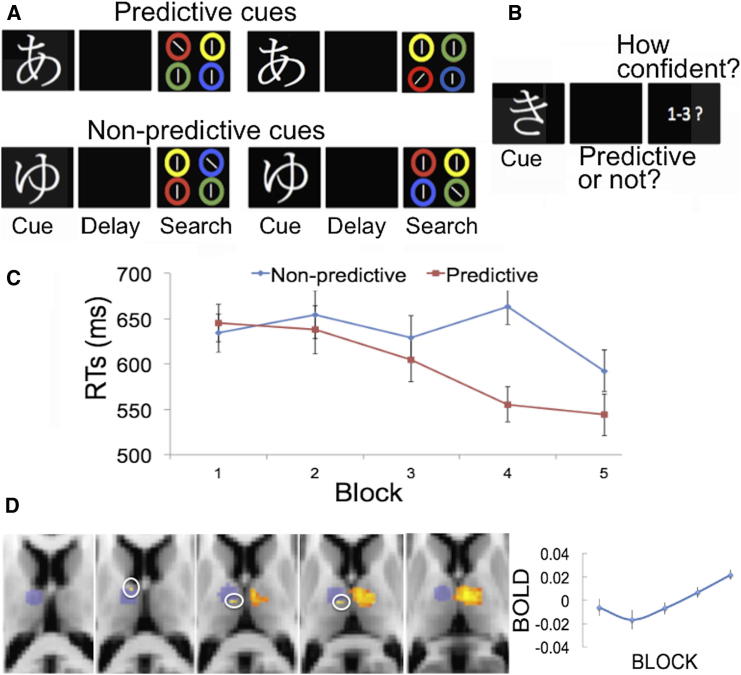
fMRI Experiment 2 (A) Learning and search phases. Participants were encouraged to form associations between the Japanese hiragana cues (not drawn to scale in the figure) and the colors of the circles surrounding the search target in order to boost search performance. The top row depicts a predictive trial (the hiragana cue is predictive of a red circle containing the tilted target). The bottom row depicts a nonpredictive trial (here, the hiragana cue is not associated with any target feature). Predictive and nonpredictive cues were presented randomly across trials. Four hiragana cues were 100% predictive (each of them was associated with a particular color surrounding the search target). Four different hiragana cues were neutral (not associated with any target features). (B) Example of a recognition test trial. To further encourage learning, we presented recognition tests following each training block, which involved the presentation of hiragana probes, and participants were required to report whether or not the probes conferred predictive value for search and to rate how confident they were in their decisions on a confidence scale of 1–3. (C) RTs for predictive cues showed evidence of learning across blocks compared to nonpredictive cues (error bars show SEM of the difference between predictive and nonpredictive RTs). Due to a technical issue, behavioral data during scanning could not be recorded for one participant. Data from the remaining 15 participants were entered into a 5 (block) × 2 (cue type: predictive and nonpredictive) repeated-measures ANOVA, which was performed over the median RTs of the correct search responses. There was a main effect of training (F(4,56) = 5.01; p = 0.006) such that search RTs became faster across the training blocks. Search performance was also faster following predictive rather than nonpredictive cues (F(1,14) = 8.77; p = 0.01). Importantly, these main effects were qualified by the presence of a significant interaction between cue type and block (F(4,56) = 4.33; p = 0.007). This interaction effect indicates that search performance became increasingly faster in predictive relative to nonpredictive trials as training developed. Search accuracy in the learning phase was very high (predictive trials = 92% correct; nonpredictive trials = 93% correct). There were no effects of block, cue, or interactions on search accuracy (for all: p > 0.45). Recognition data showed that learning of cue predictiveness improved with block (F(4,56) = 6.5; p = 0.001; Figure S4A; no other effects or interactions were evident; for all: p > 0.3). Likewise, memory confidence also increased with block (F(4,56) = 9.4; p = 0.001; Figure S4B). These results, along with the findings from the search latencies, are consistent with the acquisition of knowledge about cue predictiveness as training developed. (D) Thalamus responses followed linear and exponential trends to the predictiveness of the cues during the learning. The graph displays the percent signal change of the difference between the parameter estimate for predictive minus nonpredictive trials from all significant voxels in the right thalamus ROI across blocks.

Here, we sought to further characterize the scope of memory types that may be involved in this thalamic control of attention. In the prior patient and fMRI study, the cueing of attention was accomplished via information in WM. We devised a new fMRI paradigm (n = 16) to assess whether the thalamus mediates attention guidance that relies on the learning of new stimulus-stimulus associations and their retrieval from long-term memory. Figures 4A and 4B illustrate the paradigm.

Behavioral results were consistent with the acquisition of knowledge about cue predictiveness as training developed and its use in driving attention (Figure 4C). fMRI analyses tested for linear learning trends associated with the predictiveness of the cues (predictive > nonpredictive) across training blocks and also tested for exponential trends because the behavioral manifestation of learning had an abrupt onset in block 4 (Figure 4C). Given that we were only interested in thalamic responses, the analyses were based on anatomical ROIs comprising the entire left and entire right thalami. Responses in anterior (AV), ventrolateral (VLpd, VApc), and mediodorsal (MDpc) regions of the right thalamus (p < 0.05, corrected for multiple comparisons; Figure 4D) were consistent with both linear and exponential learning trends in the learning protocol. No clusters survived this threshold in the left thalamus. No cortical responses survived whole-brain correction.

We then conducted additional unbiased ROI analyses based on the lesion evidence from our VL thalamus patients, and, accordingly, we used a 6-mm-radius spherical ROI that covered the anatomical lesion sites of our VL thalamus patients (centered at MNI −10, −10, 10, depicted in blue shading in Figure 4D). Voxels in left VApc and on the border of VLpd and MDpc thalamus tracked the predictiveness of the hiragana cues across training, consistent with an exponential learning trend (p < 0.05, voxel corrected for multiple comparisons across patient-based ROI; Figure 4D, voxels surrounded by white circles; these ROI results were corroborated by nonparametric permutation analyses).

Although there were significant responses in the left VL thalamus, it appears that right thalamic responses were more prominent in this learning protocol relative to fMRI experiment 1. It is possible that involvement of the right thalamus is stronger when learning of stimulus-stimulus associations needs to take place to guide attention. Future studies ought to assess this possibility.

## Discussion

The present studies characterized the functional contribution of the VL and anterior regions of the human thalamus in the interplay between memory and attention. The lesion evidence demonstrates that thalamic lesions lead to impaired guidance of visual attention by WM contents. Remarkably, VL patients displayed reversed effects of cue validity independent of the relevance of the memory contents for search, even when they knew that cues were consistently associated with the target (experiments 1–3) or with a distracter (experiment 4). Importantly, the inability of the thalamic patients to use the cue in order to drive attention cannot be accounted for by an inability to retain the cue information in memory. These results are in keeping with the view that thalamic lesions disrupt the obligatory WM bias of attention that is observed in the healthy brain.

The VL thalamus, including VLa, VApc, and VLpd areas identified in our lesion and fMRI findings, is densely connected with cognitive control substrates in the prefrontal cortex (PFC) [14]. VL regions are also an integral part of the cortico-subcortical circuit comprising superior frontal regions and the superior colliculus for controlling eye movements [21, 22] and covert attention [28, 29]. This is one pathway through which the VL thalamus may be functionally relevant for the triggering of attention-biasing signals.

Furthermore, the anterior thalamus and the mamillothalamic tract are critical for normal memory function [30, 31], and the anterior nuclei also form part of a network comprising the hippocampus, mammillary bodies, and posterior cingulate cortex, which is relevant for recollective aspects of memory [15, 32]. Notably, recent research has demonstrated the role of the hippocampus in attentional guidance by WM and long-term memory [33, 34] as part of a network including the posterior cingulate and parietal cortex [34].

Based on the connectivity profile outlined above, it is possible that VL and anterior thalamus lesions lead to widespread damage of attention circuits—through connections with the superior frontal cortex and PFC [14]—and also memory circuits—via connections with hippocampus and posterior cingulate [15, 32]—which are key in controlling attention. Notably, whole-brain results from fMRI experiment 1 showed that the thalamus was coactivated along with frontoparietal areas and the hippocampus. Together, these findings help us understand the role of the thalamus in attention control by memory as part of a broader cortico-subcortical network. Thalamic damage may trigger disconnection between areas involved in perceptual selection and mnemonic control, leading to inhibition of memory-matching signals. Hence, the deployment of attention is directed away from those items.

These findings therefore indicate that in the normal functioning brain, the VL and anterior thalamus are key parts of the neural circuit mediating the automatic capture of attention by stimuli-matching WM contents, a notion that we confirmed in the first fMRI experiment.

Our lesion evidence enhances understanding of the nature of the functional role of the thalamus in memory and attention interactions beyond what could be anticipated from correlative fMRI findings alone. If the functional role of the thalamus was to regulate the activation state of memory representations based on their current relevance for task goals (e.g., down-weighting representations associated with memory distracters for search), then we would have expected thalamic lesions to produce magnified attention biases by irrelevant contents held in memory (as previously found following PFC damage [20]). PFC lesions can lead to increased attentional capture by search distracters held in WM [20], suggesting that PFC mediates the capacity to shield irrelevant WM contents from the processes that guide search. Together, these findings indicate that the thalamus’s role in WM biases of attention is dissociable from that of the PFC.

Memory biases of attention were attenuated in pulvinar patients. It has been debated whether the pulvinar’s role in attention is related to the orienting or filtering of distracters [4, 9, 35]. Our lesion evidence is consistent with a pulvinar role in the orientation of attention, namely from the contents of WM. A filtering account would have predicted exacerbated distraction by irrelevant WM contents in pulvinar patients.

Finally, our second fMRI experiment showed that thalamic responses track the acquisition of stimulus-stimulus associations that are used to optimize attention in search. Hence, it demonstrates the flexible scope of memory types supported by the thalamus in the service of attention and how this can be shaped by experience and learning.

Animal studies point to a role of the anterior thalamus in memory and learning [36–39], and human studies implicated the VL thalamus in memory and language [40–42]. These findings, together with the present work, indicate that the anterior and VL thalamus can mediate attention control driven by information held in WM that is already consolidated in the cognitive repertoire (e.g., color cues) in addition to mediating the role of experience, the learning of new regularities, and the retrieval of learned information from long-term memory to guide attention.

## Supplemental Information

Document S1. Supplemental Experimental Procedures, Figures S1–S4, and Tables S1–S4

Document S2. Article plus Supplemental Information
