# Supplementary material for: Thalamic Control of Human Attention Driven by Memory and Learning
Source: Curr Biol. 2014 May 5;24(9):993–9. doi: 10.1016/j.cub.2014.03.024 (PMC4012133; doi:10.1016/j.cub.2014.03.024)
Supplement: Document S1. Supplemental Experimental Procedures, Figures S1–S4, and Tables S1–S4 [file mmc1.pdf]

Current Biology, Volume *24*

Supplemental Information

Thalamic Control of Human Attention Driven by Memory and Learning

José de Bourbon-Teles, Paul Bentley, Saori Koshino, Kushal Shah, Agneish Dutta, Paresh Malhotra, Tobias Egner, Masud Husain, and David Soto

Supplemental Figures

Figure S1. Illustration of the lesion anatomy of the stroke patients with damage outside the thalamus, which is related to Figure 1.

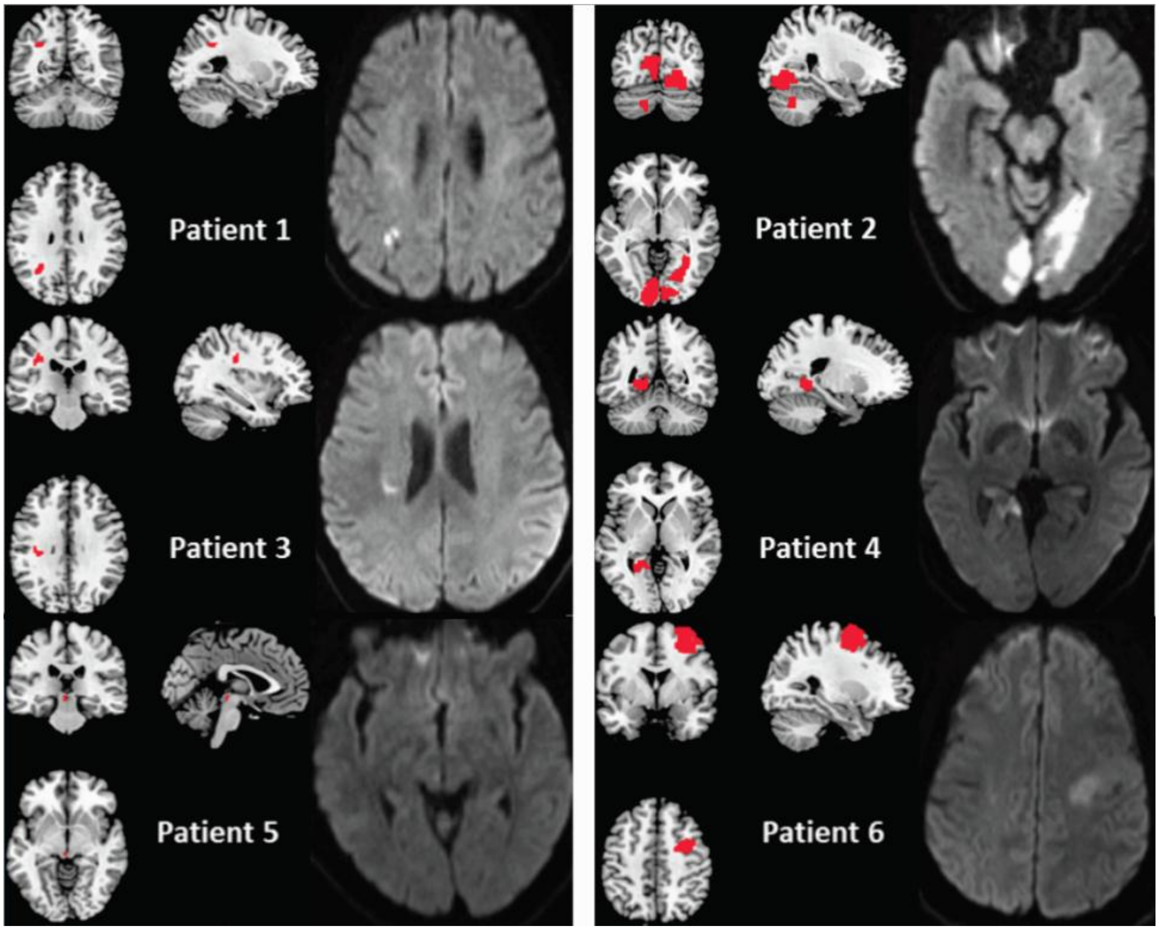

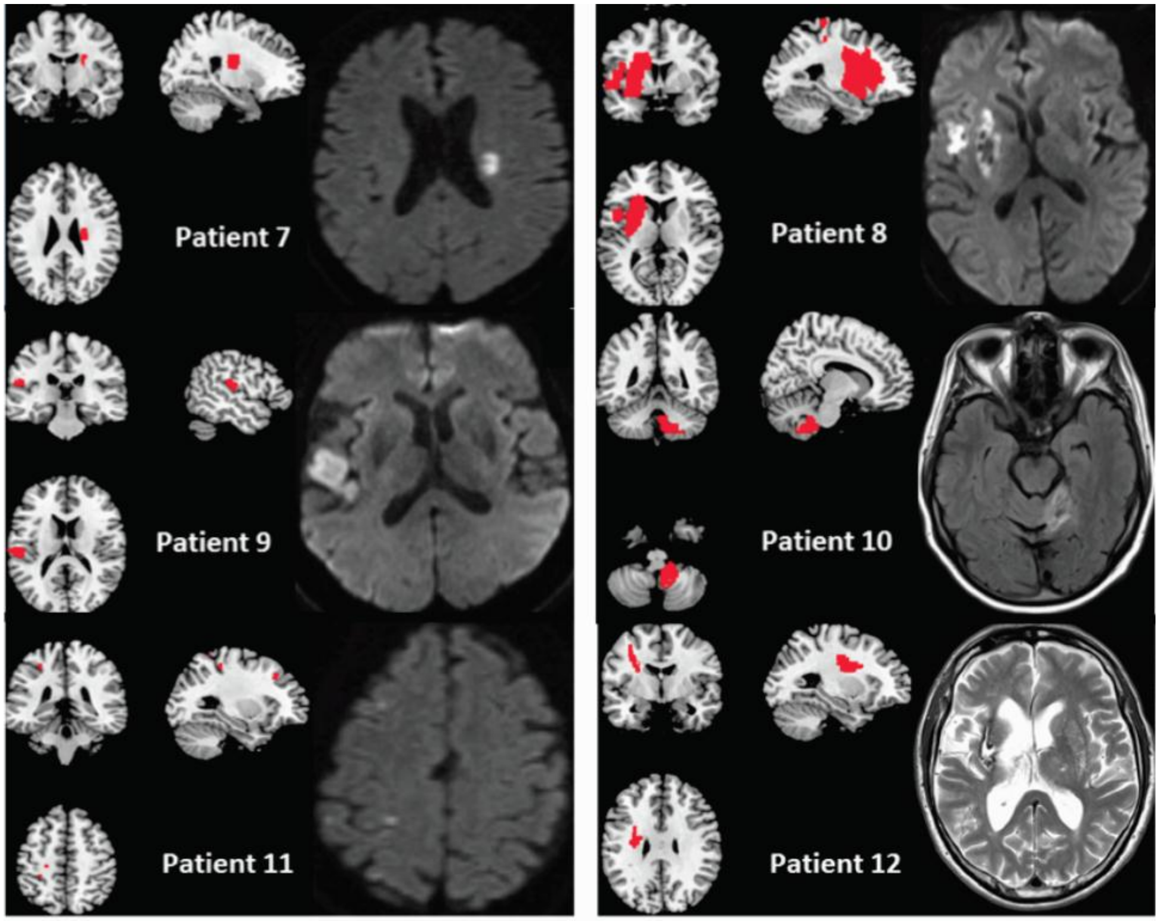
Patient 7
Patient 8
Patient 9
Patient 10
Patient 11
Patient 12

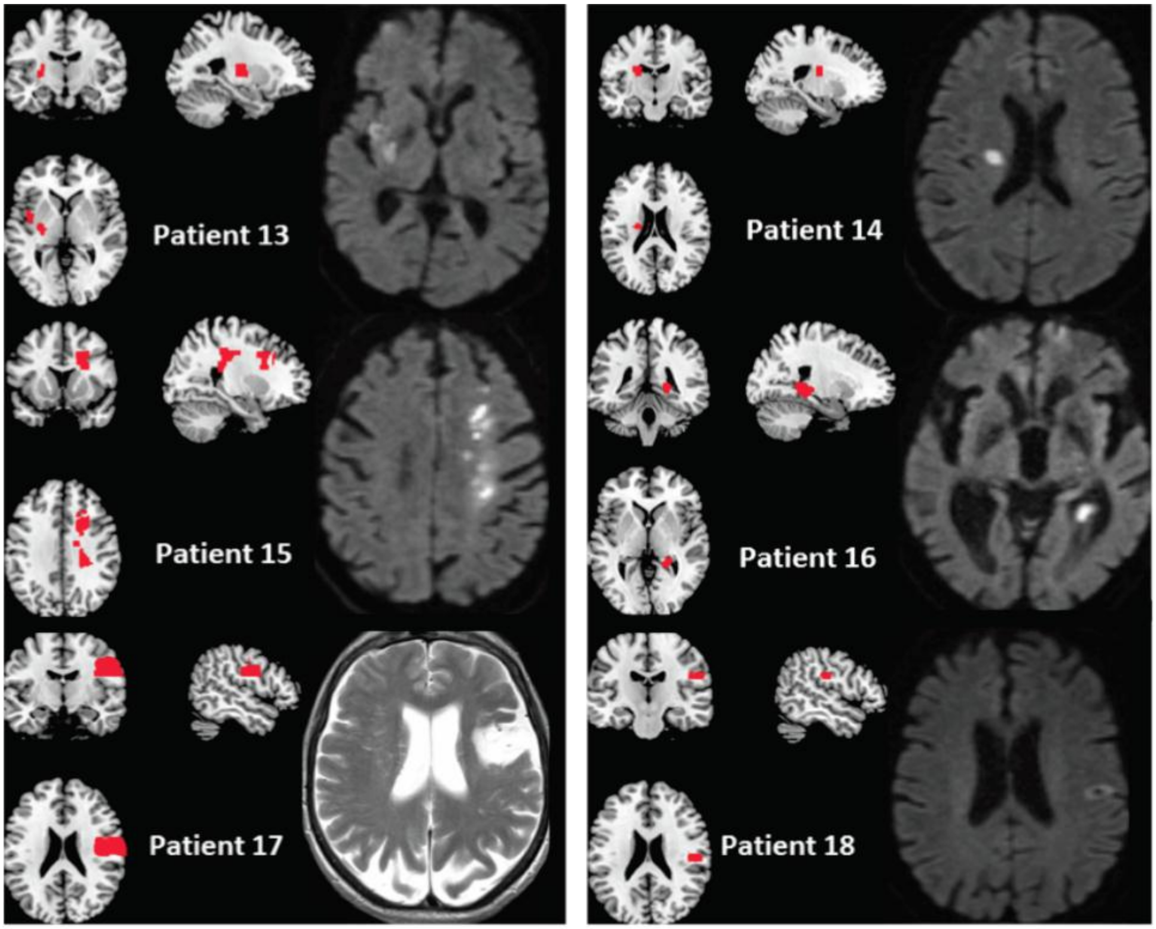
Patient 13
Patient 14
Patient 15
Patient 16
Patient 17
Patient 18

Figure S2. Behavioral data. (A) Median search reaction times in Experiment 1 as a function of cue validity (neutral in blue; valid in red) in the thalamic group, the stroke controls with lesions outside the thalamus and the non-stroke control group; this relates to Figure 2D. (B) Median Search RTs as a function of cue validity in Experiment 4 (invalid in blue, neutral in red) in the thalamic group and in the healthy age-match control group. This illustration relates to Figure 2G.

A

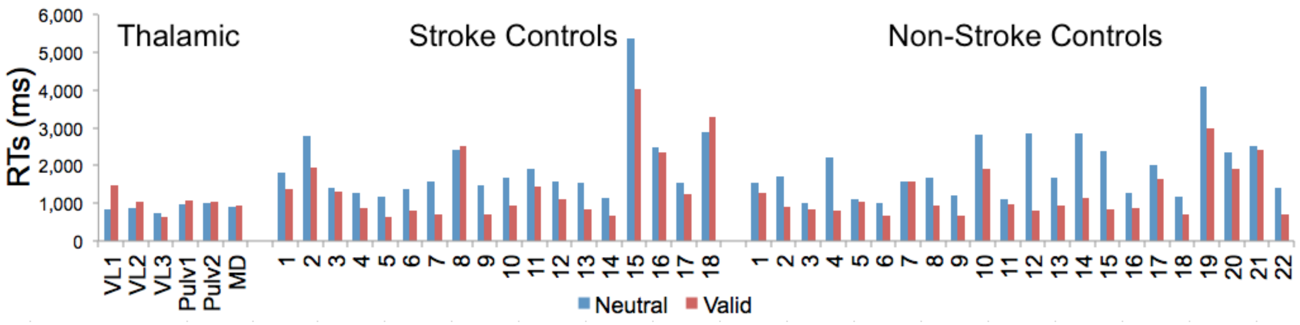

B

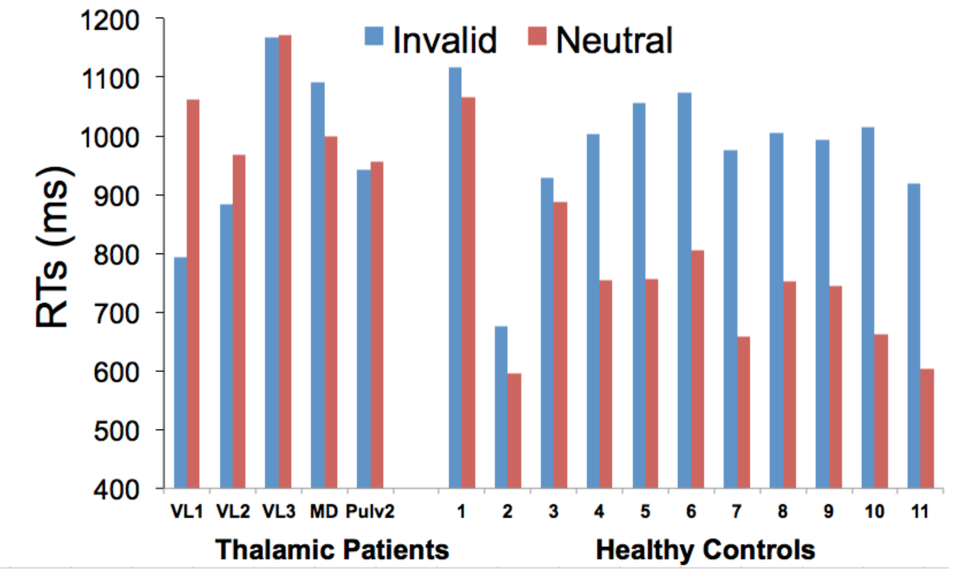

Figure S3. Reappearance effects in cortical responses, including posterior parietal cortex bilaterally, both superior (MNI: +/- 32 -54 50) and inferior parietal lobe bilaterally (MNI: +/-60 -30 30), and left rostral PFC (MNI: 20, 58, 0). This illustration is related to Figure 3C.

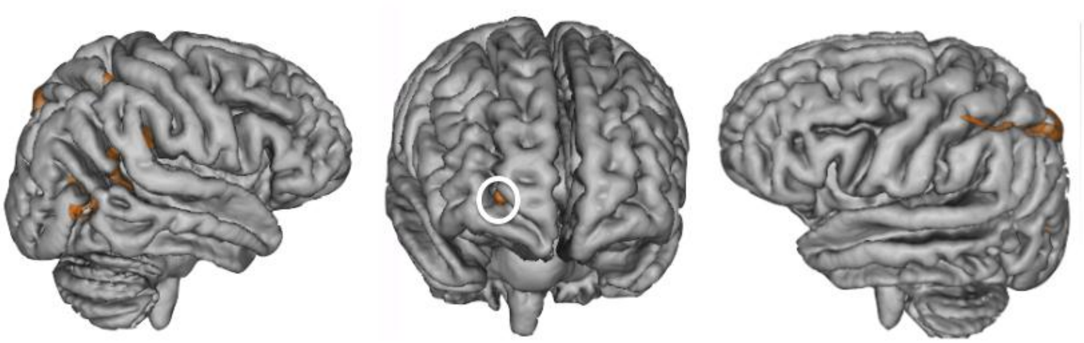

Figure S4. Recognition memory performance for the predictiveness of the hiragana cues (A) Memory accuracy (B) Confidence ratings (error bars= s.e.m.) related to Figure 4C.

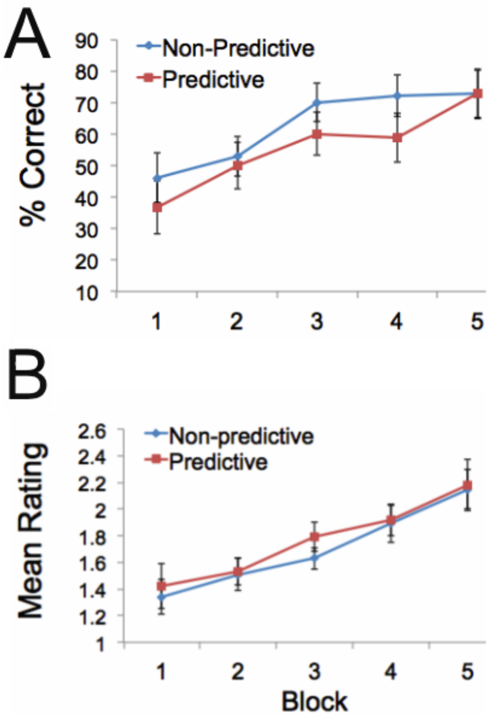

Supplemental Tables

Table S1. Percentage of thalamus nucleus occupied by the patient's lesion and cortical connectivity information. Thalamic nuclei are based on histology-based atlas [1].

Abbreviations:
Medial: MD: Mediodorsal nucleus | **MDmc:** Magnocellular part | **MDpc:** Parvocellular part | **CL**: Central lateral nucleus | **CeM:** Central medial nucleus | **CM:** Centromedian nucleus | **Pf:** Parafascicular nucleus

Posterior: **PuM:** Pulvinar Medial | **PuI:** Inferior | **PuL**: Lateral | **PuA**: Anterior | **LP**: Lateral posterior nucleus | **MGN**: Medial geniculate | **SG**: Suprageniculate nucleus | **Li**: Limitans nucleus

Lateral: **VPLa**: Ventral posterior lateral, anterior part | **VPLp**: posterior part | **VPM**: Ventral posterior medial nucleus | **VPI**: Ventral posterior inferior nucleus | **VLa**: Ventral lateral anterior nucleus | **VLpd:** Ventral lateral posterior dorsal | **VLpv:** posterior ventral | **VAmc**: Ventral anterior magnocellular | **VApc**: parvocellular part | **VM**: Ventral medial

Anterior: **AD:** Anterior dorsal | **AM:** Anterior medial | **AV**: Anterior ventral | **LD:** Lateral dorsal.

Cortical connectivity information
The superscripts indicate the cortical region that each damaged thalamic nuclei projects to according to the Oxford thalamic connectivity atlas derived from diffusion tensor imaging and probabilistic tractography [2].

temp: temporal | **pfc**: prefrontal | **ppc**: posterior parietal | **occ**: occipital | **premot**: premotor | **mot**: motor | **ss**: somatosensory

Patient

	VL1	VL2	VL3	MD	Pulv1	Pulv2
AD ^{temp}			44.4			
AM ^{temp}			1.0			
AV ^{temp}			92.5			
CL $^{temp\ pfc}$			48.0	63.8	71.4	3.5
CM ^{pfc}			39.3	1.0	73.4	55.8
CeM $^{temp\ pfc}$			94.2	7.5	9.8	
LD ^{temp}			53.7		29.9	
LP ^{ppc}		3.1	33.5	6.6	35.9	46.0
Li $^{pfc,\ ppc}$				68.3	68.3	6.2
MDmc $^{temp\ pfc}$			49.8	48.3	31.1	
MDpc ^{pfc}			66.0	78.8	75.4	3.3
MGN ^{ppc}					31.1	77.9
Pf ^{pfc}			27.9	89.6	41.5	21.9
Po $^{ss\ \ ppc}$				32.4	97.2	84.6

PuA $^{\text{ss mot ppc}}$		1.2	24.6	45.0	1.0	94.6
PuI $^{\text{ppc occ temp}}$					14.5	1.0
PuL ^{ppc}					78.8	91.8
PuM $^{\text{ppc occ temp pfc}}$				5.2	58.4	58.4
SG ^{ppc}				13.8	55.2	39.0
VAmc ^{pfc}		2.3	1.0			
VApc ^{pfc}	32.4	38.5	1.0			
VLa ^{pfc}	77.4	94.7	1.0	3.7	1.4	
VLpd ^{pfc}	3.8	33.9	1.0	5.2	32.9	
VLpv ^{pfc}	21.9	5.9	99.5	28.4	72.0	7.5
VM ^{pfc}	1.3	35.9	99.4	47.2	23.3	1.3
VPl ^{ss}		1.1	23.2		1.0	1.0
VPLa $^{\text{ss premot}}$	3.4	92.4	1.0		1.0	44.4
VPLp ^{ss}		29.5	8.7	14.6	96.0	56.6
VPM ^{pm}		8.6	61.8	6.2	86.2	76.3

Table S2. Median RTs and s.e.m across the different validity conditions in (A) Experiment 1 and (B) Experiment 4.

(A)

	Median RT		s.e.m	
	**Valid**	**Neutral**	**Valid**	**Neutral**
VL1	1482.00	833.50	81.54	90.33
VL2	1038.00	883.00	64.44	53.42
VL3	651.00	726.50	17.57	16.24
Pulv1	1069.00	971.00	51.57	46.35
Pulv2	1047.00	966.50	33.82	23.08
MD	922.00	917.00	33.98	27.96

(B)

	Median RT		s.e.m	
	**Invalid**	**Neutral**	**Invalid**	**Neutral**
VL1	792.50	1062.00	18.53	25.4
VL2	882.50	967.00	26.98	31.15
VL3	1168.00	1171.00	18.93	17.94
Pulv2	1091.00	999.00	26.48	22.06
MD	943.00	956.00	24.14	19.01

Table S3. Control group of patients with lesion outside the thalamus. Demographic description: sex/age, main lesion site. Most patients had ischemic stroke except patients 3 and 6 who were hemorraghic, and patient 8 who was both ischemic and hemorraghic.

**Patients**	**Sex/Age**	**Main Lesion Site**
1	Female/70	Left parietal
2	Male/74	Occipital and cerebellum
3	Male/58	Left white matter
4	Male/70	Left occipital lobe
5	Male/28	Midbrain
6	Male/66	Right superior frontal gyrus
7	Male/59	Right corona radiata
8	Male/59	Left insula, striatum and parietal
9	Female/66	Left temporoparietal
10	Male/55	Right cerebellum
11	Male/64	Left frontoparietal
12	Male/58	Left insula and right white matter
13	Male/74	Left insula, striatum
14	Male/51	Left striatum
15	Male/70	Right frontoparietal
16	Male/82	Right hippocampus
17	Male/68	Right pre/postcentral gyrus
18	Male/79	Right postcentral gyrus

Table S4. Performance of the thalamic patients in the Addenbrooke's Cognitive Examination (ACE) and Digit Span tests.

**Tests**	**Addenbrooke's Cognitive Examination (ACE)**						**Digit Span**	
Patients	Attention and Orientation	Memory	Fluenc y	Languag e	Visuo-spatial	Total ACE-R score	Forward	Backwar d
VL1	18/18	23/26	11/14	25/26	16/16	93/100	13/16	12/14
VL2	17/18	21/26	8/14	22/26	15/16	83/100	13/16	8/14
VL3	18/18	16/26	6/14	23/26	16/16	79/100	13/16	8/14
Pulv2	18/18	24/26	11/14	26/26	15/16	94/100	12/16	9/14
MD	18/18	22/26	9/14	24/26	11/16	84/100	11/16	4/14

Supplemental Experimental Procedures

Patient Studies

Participants

Six patients with thalamic lesions due to stroke, 18 patients with lesions outside the thalamus and 22 non-stroke patients were recruited at Charing Cross Hospital (London). The study was approved by the West London Research Ethics committee.

Patients VL1 (Male/74 years), VL2 (Female/59 years) and Pulv1 (Male/80 years) were initially tested in their acute stage in Experiment 1. Patient Pulv1 received no further testing whereas patients VL1 and VL2 received further testing up to 1 year following stroke. VL3 (Male/70 years) was recruited to take part in Experiment 1 and 4 five years after stroke and testing occurred in different sessions in separate days. Pulv2 (Male/68 years) and MD (Male/78 years) were tested in different sessions 3 and 10 months after stroke (in Experiment 1 and 4). All of them were right handed and suffered from ischemic stroke. Table S3 illustrates demographic and lesion site information for the control group of stroke patients and Fig. S1 depicts the anatomical site of their lesions.

The thalamic patients also completed the Addenbrooke's cognitive examination and standard forward and backward Digit Span tests test to gauge an indication of general cognitive ability (Table S4).

Task and Procedure

The tasks were programmed using E-Prime v2.0 (Psychology Software Tools Inc., Pittsburgh, USA; www.pstnet.com/eprime.cfm). Participants provided written informed consent to take part in the experiments. Prior to each task they received instructions on the validity of the cues for search and the relevant response mappings. Patients received practice trials until they felt comfortable performing the task.

Each search trial started with the presentation of a word (i.e. "Valid" or "Neutral") indicating whether or not the upcoming search trial would be validly cued (Experiment 1, 2, 3) to ensure the patients had foreknowledge of the cue validity prior to search. Valid and neutral trials were randomly presented. The experimenter then started the trial via

button press. A fixation point appeared for 1 s. Then the verbal cue (i.e. Red, Green, Blue, Yellow or Pink) appeared for 3 s. In the neutral condition the text “No cue” was displayed. Cue offset was followed by a 2 s delay (or 6 sec in Experiment 3) with a blank screen, which was then followed by the search display. There were four distinctive colored outline circles, each drawn any of five hues. Three of the circles had a gap either on the top-left or the bottom-right. The search target had two gaps, one on the top and on the bottom. The task was to locate the target circle (i.e. pressing one button for targets in the left side of the display and another button for right targets).

On valid trials (Experiments 1, 2 and 3), the verbal cue always specified the color of the search target and patients were instructed to keep that information online to maximize search performance. Because the word cues were consistently associated with the search target, they acted as ‘search template’, which according to extant theories of attention must be held in working memory (WM) to guide search [3]. Notably, given that the cue varied from trial to trial, there was indeed need for updating the content of the verbal cue in WM in order to drive attention. Further we elected to use color word cues in the Patient Study rather that colored shapes to avoid the presence of lower-level sensory priming effects. Finally, we note that our prior work in healthy subjects consistently indicates that verbal cues, like the ones used here, need in fact to be committed to WM in order to bias attention [4, 5].

On invalid trials (Experiment 4), the verbal cue never specified the color of the search target and rather the memory cue always matched a search distracter when reappeared in the search display. Participants received clear instructions about these contingencies and were encouraged to optimize search for the target circle with the 2 vertical gaps.

In addition to the search trials, Experiments 1 and 2 also included a recognition test in order to gauge the patient’s ability to keep the cues in memory. Note that the recognition test in Experiment 3 and 4 was embedded in the search trials and followed each search response while in Experiment 1 and 2 search and memory recognition testing were performed in separate blocks of trials (see below).

Each trial of the recognition test presented the observer with a color word for 3 s. Following a delay of 2 s with a blank screen, a recognition memory test appeared. Here a colored circle appeared and the task was to report whether or not the color word matched the circle's color. Patients gave their responses aloud, which were then recorded by the experimenter by pressing a different button for 'same' and 'different' responses.

The number of trials in Experiment 1 depended on patient's availability; patients VL1, VL2 and Pulv1 performed one block of 40 trials (20 valid and 20 neutral) except patients VL3, Pulv2 and MD who performed a total of 120 trials (60 valid and 60 neutral). Patients VL1, VL2, VL3 and Pulv1 performed the search task in Figure 2A and in addition performed 12 trials in the delayed memory test (Figure 2B). Patients Pulv2 and MD performed the same task but including a memory test for the initial cue at the end of each valid search trial. This was also due to time constrains in testing these patients. They were recruited in the chronic stage and we wished to ensure from the outset they attempted to commit the cue in WM throughout the trials. Patients VL1 and VL2 were subsequently tested in the same conditions and their data is presented as a control Experiment 3 to confirm that their unique performance impairment was not due to inability to remember the cue. Data from all the thalamic patients was presented together as Experiment 1 to illustrate the performance impairment of the thalamus group relative to the controls.

Experiment 2 involved the same task as Experiment 1 with the exception that the delay period between cue and search display was manipulated (i.e. short delay: 2 sec vs. long delay: 6 sec; randomly selected across trials and with equal probability of occurrence). Patients completed 128 trials with 32 trials per each validity and delay condition. Each patient also performed the 12 trials in the delayed memory task.

Experiment 3 was similar to Experiment 1 except that the recognition test was embedded in the search trials and therefore memory probes followed each search response. There were 60 trials (30 valid and 30 neutral trials; randomly selected.

In Experiment 4, the memory cue was always invalid when it reappeared in the search display. There were 120 trials (60 invalid and 60 neutral).

fMRI Studies

Experiment 1

Participants

Thirty-nine participants took part (20 females; age range: 21-23 years) in return for £20. They were native-English speakers and naive with regard to the experimental aims and hypothesis. No participant had a prior history of neurological or neuropsychiatric disorders, and all had normal vision. This fMRI experiment is part of a bigger study for which DTI and resting state BOLD data were collected and that will be reported in a separate paper.

Task and Procedure

Each trial began with a fixation display for 0.5 sec that was followed by 1 s delay before the onset of the memory cue. The cue appeared for 0.25 s and was a colored circle drawn in one of four different colors. The offset of the cue was followed by a delay period of 1 s. Then, the search display was presented for 0.1 s and was composed of three colored circles at the corners of an imaginary triangle. Each circle contained a line; two of the lines were vertical while the target line was tilted 16° either to the left or right. The participants' task was to indicate the orientation of the tilted line via button press during a time window of 1 s. Target location and orientation were randomly selected. The memory and search circles were randomly chosen from one of four colors (red, blue, green, or yellow). Each circle was a color singleton in the search display. The cues could be ’invalid’ or ‘neutral’ depending on whether they surrounded a search distracter or did not reappear in the search display. To ensure that any WM effect on search occurred automatically, namely, without intention on the part of the observer (cf. obligatory coupling between memory and attention) it is fundamental to avoid the contribution of top-down strategic factors (i.e. expectancy that the contents of WM may be associated with the search target) [6, 7]. Accordingly, we made sure participants were fully aware of the invalidity of the memory cues for search by blocking the invalid and neutral conditions. There were 6 invalid and 6 neutral blocks (Inv-Neu-Inv-Neu-Inv-Neu-Inv-Neu-Inv-Neu-Inv-Neu) with each including 10 trials (8 search and 2 memory catch trials, see next). We ensured that participants held the cues in WM throughout the trials by using memory “catch” trials (20%) where a memory probe was presented for 1.5 s instead of the visual search display. The probe consisted of a colored circle, and

participants had to provide a match/mismatch response, indicating whether or not the probe was the same color as the memory cue.

Participants received a 5 s instruction display at the beginning of each block, which informed about the nature of cue in the subsequent trials. Participants received clear instructions about the presence of memory “catch” trials. Participants completed several practice sessions outside the scanner, initially with a visual search display duration of 0.5 s followed by a search display duration of 0.1 s until performance was above 90% correct. They were told to avoid eye movements during the search, and we used the very brief search display time (0.1 s) to discourage eye movements.

Scanning Parameters

We used a Siemens Avanto 1.5T MRI scanner and a 32-channel head coil. Sagittal T2-weighted volumes were acquired (220 volumes) with a field of view (FOV) of 205 × 205 mm, repetition time (TR) of 2.5 s, echo time (TE) of 44 ms, and slice thickness of 3.2 mm. A six minutes T1-weighted structural scan was also collected.

Data Analysis

We used FEAT (fMRI Expert Analysis Tool) Version 6.0, as part of FSL (www.fmrib.ox.ac.uk/fsl). The first four EPI volumes were removed to account for T1 equilibrium effects. Non-brain removal was performed using Brain Extraction Tool [8]. Motion correction of functional scans was carried out using FMRIB’s Linear Image Registration Tool [9]. We applied a 100 s high-pass temporal filtering to remove low frequency noise, and spatial smoothing using a FWHM Gaussian kernel of 6 mm. Time-series statistical analyses were conducted using FILM (FMRIB’s Improved Linear Model) with local autocorrelation correction [10]. The data were analyzed using voxelwise time series analysis within the framework of the general linear model. A design matrix was generated with a double-gamma hemodynamic response function and its first temporal derivative; invalid and neutral blocks were modelled as regressors of interest. The 5 s instruction prior to each block and the presence of catch trials and error responses were modelled as regressors of no interest. We obtained contrasts of parameter estimates for invalid > (<) neutral for each individual, which were carried forward to a higher-level analysis using FLAME 1+2 (FMRIB’s Local Analysis of Mixed Effects) in the form of one-sample t-test to assess consistent regions of activation across participants. The

data were registered to individual high-resolution structural images using FLIRT Boundary-Based Registration [11], and then co-registered into standard MNI space. We report thalamic maps of BOLD responses thresholded using clusters determined by a voxelwise threshold of Z = 2.3(P<0.01) and a cluster significance threshold of P=0.05, corrected for multiple comparisons (using Gaussian Random Field Theory) within the left and right thalamus ROIs [12]. For the more exploratory analyses of cortical activations we followed Hayasaka and Nichols [13] and employed a more stringent voxelwise cluster-forming threshold of Z>3.09 (P<0.001) along with a cluster significance threshold of P=0.05, corrected for multiple comparisons across the whole brain.

Experiment 2

Participants

Sixteen new right-handed healthy participants (seven females; age range: 18-31 years) participated in the study in return for £20. Due to a technical problem behavioural data from one participant could not be recorded.

Task and Procedure

Learning study phase

Each trial of the learning phase began with the presentation of one of eight possible Japanese hiragana symbols (the cues) for 0.5 s. Then, a blank screen was presented for 1.5 s, which was followed by a search display comprising four outline circles in red, green, yellow and blue displayed at the corners of an imaginary square. Three of the colored circles contained a vertical line and the remaining circle contained a tilted line (the search target). The search task was the same as in fMRI Experiment 1. Search display appeared for 0.1 s to discourage eye movements. Four of the hiragana cues were predictive of the color of the circle surrounding the search target; each of the 4 predictive hiragana cues was associated with one of the four colors. The remaining 4 hiragana cues were non-predictive and not associated with any target feature. The color of the circle surrounding the search target was randomly selected on these non-predictive trials. The inter-trial time interval (ITI) was jittered between 2.5 and 4 s, with a pseudo-exponential distribution (50% of ITIs were 2.5 s, 25% of ITIs were 3 s, 12.5% were 3.5 s, and 12.5% were 4 s), to facilitate the independent estimation of BOLD responses across trials. A central fixation point was presented during the ITI. The task

was composed of 5 ‘search’ blocks of 16 trials each (8 predictive and 8 non-predictive; each symbol was displayed 2 times per block randomly across trials).

Each ‘search’ block was followed by a recognition test to ensure that participants were indeed attempting to learn the predictive value associated with the different hiragana cues. Each recognition block began with a 2.5 s instruction display. On each trial, participants were presented with one of the hiragana cues for 0.5 s, followed by a blank screen for 2.5 s during which participants had to indicate via button press whether the symbol was predictive or non-predictive. Following this, participants were asked to rate how confident they were of their decision during a time window of 1.5 s. A 1-3 rating scale was used (1: no confidence; 2: medium confidence; 3: high confidence). Each recognition block comprised 8 trials, one for each of the hiragana cues used (4 predictive and 4 non-predictive).

Prior to scanning, participants received instructions and training on the task. Note that the hiragana cues used in the training phase were different from the ones used in the scanner. Participants were explicitly trained to maintain central fixation throughout the trials and to avoid eye movements like in fMRI Experiment 1.

Scanning Parameters

These were identical to fMRI Experiment 1 except that 342 volumes were acquired here. The duration of the scan was based on pilot testing which determined the amount of exposure needed to observe learning effects.

Data Analysis

The analysis pipeline was identical to Experiment 1 unless otherwise noted. The trials of the learning protocol were modelled from the onset of the hiragana symbols with duration of 2.1 s (including 0.5 s cue exposure, 1.5 s blank screen delay and 0.1 s search display). We separately modelled the predictive and non-predictive cues for each of the five learning blocks independently. The recognition trials were modelled from the onset of the memory probes (predictive and non-predictive) for each of the five recognition blocks separately. We also included regressors for the onset of the confidence rating periods. Finally we modelled the search error trials and the instruction display periods preceding each of the search and recognition test phases.

We obtained contrasts of parameter estimates for predictive > (<) non-predictive trials, which were carried forward to second-level within-subject analysis (fixed effects) to test for linear and exponential trends across the 5 blocks. Finally, we performed group-level analyses as in fMRI Experiment 1. Co-registration of the functional data to standard space and the statistical thresholding of the functional activations in the thalamus ROI analyses were also performed as in Experiment 1. Additional unbiased ROI analyses based on the area of overlap of the VL patient lesions were also performed using a 6 mm radius sphere; we employed parametric tests and voxelwise correction for multiple comparisons ($P<0.05$) using GRF-theory-based maximum height threshold, and also nonparametric permutation testing to further corroborate the results. Five thousand random permutations were calculated to create the null distribution for assessing the test statistics.

Supplemental Analyses

Patient Study

Experiment 1

To confirm that the results held when the data were transformed to account for variation in RT across individuals, we computed the individual cuing effect as (Neutral RT – Valid RT)/(Neutral RT - Valid RT). Analyses confirmed that thalamic patients had lower cuing effect relative to patients with lesions outside the thalamus ($t(22)=-4.01$, $p=0.001$) and the non-stroke group ($t(26)=-4.41$, $p=0.0001$), which did not differ ($p>0.343$).

Experiment 4

We computed the individual cue invalidity effect as (Invalid RT - Neutral RT)/(Invalid RT - Neutral RT) and confirmed the size of the invalidity effect in the thalamus patients was significantly lower than in the healthy controls ($t(14)=-4.374$, $p=0.0001$).

Supplemental Data

Replication of the ‘reversed’ validity effect

Patients VL1 and VL2 took part in this replication experiment conducted one month later in which we also varied the delay between memory and search. There were 64 trials (32 valid and 32 neutral). The same reversed validity effect was evident, with faster search RTs in neutral relative to valid conditions. Mean search accuracy was high (VL1: Neutral: 97%, Valid: 81%; VL2: Neutral: 100%, Valid: 100%).

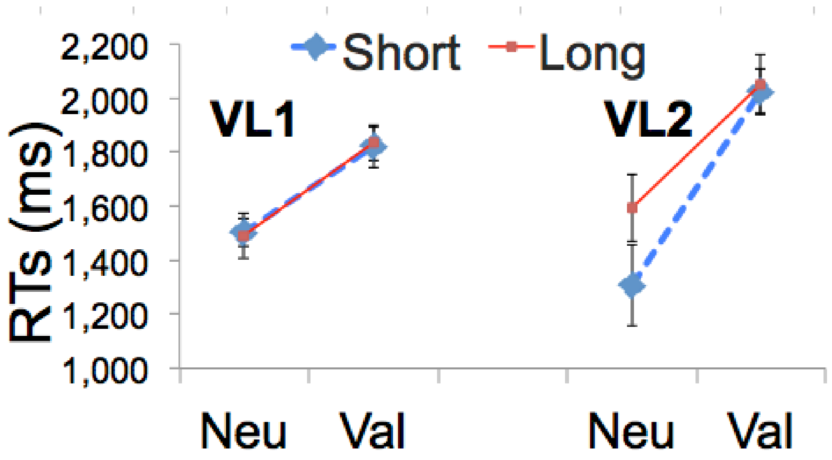

Supplemental References

S1. Krauth, A., Blanc, R., Poveda, A., Jeanmonod, D., Morel, A., and Szekely, G. (2010). A mean three-dimensional atlas of the human thalamus: generation from multiple histological data. Neuroimage *49*, 2053-2062.

S2. Behrens, T.E., Johansen-Berg, H., Woolrich, M.W., Smith, S.M., Wheeler-Kingshott, C.A., Boulby, P.A., Barker, G.J., Sillery, E.L., Sheehan, K., Ciccarelli, O., et al. (2003). Non-invasive mapping of connections between human thalamus and cortex using diffusion imaging. Nat Neurosci *6*, 750-757.

S3. Olivers, C.N., Peters, J., Houtkamp, R., and Roelfsema, P.R. (2011). Different states in visual working memory: when it guides attention and when it does not. Trends Cogn Sci *15*, 327-334.

S4. Soto, D., and Humphreys, G.W. (2008). Stressing the mind: the effect of cognitive load and articulatory suppression on attentional guidance from working memory. Percept Psychophys *70*, 924-934.

S5. Soto, D., and Humphreys, G.W. (2007). Automatic guidance of visual attention from verbal working memory. J Exp Psychol Hum Percept Perform *33*, 730-737.

S6. Woodman, G.F., and Luck, S.J. (2007). Do the contents of visual working memory automatically influence attentional selection during visual search? J Exp Psychol Hum Percept Perform *33*, 363-377.
S7. Soto, D., Heinke, D., Humphreys, G.W., and Blanco, M.J. (2005). Early, involuntary top-down guidance of attention from working memory. J Exp Psychol Hum Percept Perform *31*, 248-261.
S8. Smith, S.M. (2002). Fast robust automated brain extraction. Hum Brain Mapp *17*, 143-155.
S9. Jenkinson, M., Bannister, P., Brady, M., and Smith, S. (2002). Improved optimization for the robust and accurate linear registration and motion correction of brain images. Neuroimage *17*, 825-841.
S10. Woolrich, M.W., Ripley, B.D., Brady, M., and Smith, S.M. (2001). Temporal autocorrelation in univariate linear modeling of FMRI data. Neuroimage *14*, 1370-1386.
S11. Greve, D.N., and Fischl, B. (2009). Accurate and robust brain image alignment using boundary-based registration. Neuroimage *48*, 63-72.
S12. Worsley, K.J. (2001). Functional MRI: An Introduction to Methods, (Oxford University Press.).
S13. Hayasaka, S., and Nichols, T.E. (2003). Validating cluster size inference: random field and permutation methods. Neuroimage *20*, 2343-2356.
