# Supplementary material for: Thalamic Control of Human Attention Driven by Memory and Learning
Source: Curr Biol. 2014 May 5;24(9):993–9. doi: 10.1016/j.cub.2014.03.024 (PMC4012133; doi:10.1016/j.cub.2014.03.024)
Supplement: Document S2. Article plus Supplemental Information [file mmc2.pdf]

Current Biology 24, 993–999, May 5, 2014 ©2014 The Authors http://dx.doi.org/10.1016/j.cub.2014.03.024

Report

Thalamic Control of Human Attention Driven by Memory and Learning

José de Bourbon-Teles,[1] Paul Bentley,[1] Saori Koshino,[1] Kushal Shah,[1] Agneish Dutta,[1] Paresh Malhotra,[1] Tobias Egner,[2] Masud Husain,[3] and David Soto[1,*]
[1]Division of Brain Sciences, Department of Medicine, Imperial College London, Charing Cross Campus, St. Dunstan's Road, London W6 8RP, UK
[2]Center for Cognitive Neuroscience and Department of Psychology & Neuroscience, Duke University, Levine Science Research Building, Box 90999, 450 Research Drive, Durham, NC 27708, USA
[3]Department of Experimental Psychology, University of Oxford, South Parks Road, Oxford OX1 3UD, UK

Summary

The role of the thalamus in high-level cognition—attention, working memory (WM), rule-based learning, and decision making—remains poorly understood, especially in comparison to that of cortical frontoparietal networks [1–3]. Studies of visual thalamus have revealed important roles for pulvinar and lateral geniculate nucleus in visuospatial perception and attention [4–10] and for mediodorsal thalamus in oculomotor control [11]. Ventrolateral thalamus contains subdivisions devoted to action control as part of a circuit involving the basal ganglia [12, 13] and motor, premotor, and prefrontal cortices [14], whereas anterior thalamus forms a memory network in connection with the hippocampus [15]. This connectivity profile suggests that ventrolateral and anterior thalamus may represent a nexus between mnemonic and control functions, such as action or attentional selection. Here, we characterize the role of thalamus in the interplay between memory and visual attention. We show that ventrolateral lesions impair the influence of WM representations on attentional deployment. A subsequent fMRI study in healthy volunteers demonstrates involvement of ventrolateral and, notably, anterior thalamus in biasing attention through WM contents. To further characterize the memory types used by the thalamus to bias attention, we performed a second fMRI study that involved learning of stimulus-stimulus associations and their retrieval from long-term memory to optimize attention in search. Responses in ventrolateral and anterior thalamic nuclei tracked learning of the predictiveness of these abstract associations and their use in directing attention. These findings demonstrate a key role for human thalamus in higher-level cognition, notably, in mnemonic biasing of attention.

Results

We assessed a group of thalamic patients in a task probing the interaction between working memory (WM) contents and visual attention [16, 17], and then we used fMRI in healthy volunteers to test predictions derived from the lesion data. The study was approved by the West London Research Ethics committee.

Patient Study

Lesion maps appear in Figure 1. Figures 2A–2C depict the experimental paradigm (see also Supplemental Experimental Procedures available online). Thalamic patients' performances were compared to 18 stroke controls (see Figure S1 for lesions) and 22 subjects without stroke who were admitted to the hospital for neurological evaluation.

Experiment 1

We tested whether thalamic lesions disrupt WM cueing effects on search. Analyses of cue validity effects (neutral reaction time [RT] − valid RT) showed that control groups used the cues strategically to boost search. Thalamic patients consistently failed to do so, showing reduced validity effects relative to controls (Figure 2D; for statistics, see figure legend). Results held when cueing effects were transformed in order to account for interindividual variation in reaction time (RT) (i.e., (neutral RT − valid RT)/(neutral RT + valid RT); see Supplemental Experimental Procedures). No effects of target visual field (i.e., contralesional versus ipsilesional) were found here or in subsequent experiments.

Intriguingly, two ventrolateral (VL) patients displayed a "reversed" validity effect, with slower performance in valid versus neutral trials. Patient VL3 showed a validity effect in the "normal" direction; however, its size was reduced relative to controls. We confirmed that validity effects in the control groups were higher than they were in each individual thalamic patient (all one sample $t > 4$, $p < 0.0001$; see also Figure S2A for individual data).

Notably, patients VL1 and VL2 were tested in the acute stroke phase in the present experiment and subsequently also in the chronic phase (up to 1 year later; see experiments 2–4). Testing in both acute and chronic phases mitigated the possibility that our findings relate to functional or maladaptive plasticity. Patient VL3, however, took part in the first experiment 5 years poststroke. Hence, due to this longer recovery period, it is likely that compensatory mechanisms may have operated to regain some of the functional loss in this patient.

Importantly, delayed recognition performance was high in the thalamic group (94.6% correct) and in the two control groups (95.4% and 94.4% correct, respectively) with no difference among control groups (for all: $p > 0.8$). The inability of thalamic patients to use the cue to guide search could thus not be explained by inability to retain it.

Experiment 2

Given the striking reversed validity effect in patients VL1 and VL2, we sought to replicate this in experiment 2. Patients were reexamined 6 months and 10 months after experiment 1, respectively (Figure 1; bottom row confirms the chronic stage of VL lesions). We also varied the delay (2 s versus 6 s) between cue and search displays to assess whether the absence of a cueing effect in experiment 1 could be improved by allowing the patients to have more time to use the cue.

Again, VL patients showed a reversed validity effect, which was not modulated by the delay between cue and search

*Correspondence: d.soto@imperial.ac.uk
This is an open access article under the CC BY license (http://creativecommons.org/licenses/by/3.0/).
CrossMark

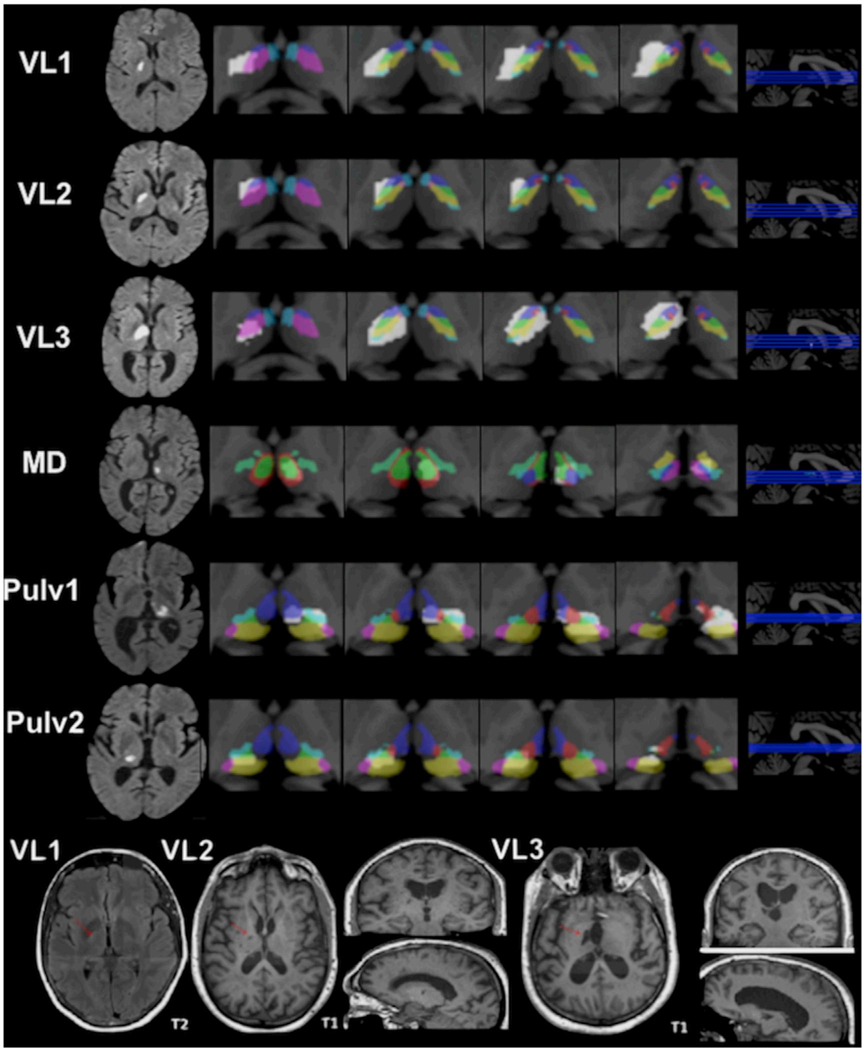

Figure 1. Lesion Maps of Thalamic Patients

For the sake of simplicity, patients (n = 6) are labeled by the name of the thalamic lesion site showing overlap across them. Diffusion weighted imaging (DWI) scans (left column) illustrate the thalamic lesion site in the acute stage. Individual lesions were mapped onto a 3D, high-resolution, histology-based atlas of thalamic areas [18]. Axial slices to the right depict the thalamic nuclei in different colors, and the lesion area is highlighted in white. Table S1 depicts the percentage of damage of each thalamic nucleus and connectivity information from a diffusion tensor imaging atlas of probabilistic connections between thalamic nuclei and cortical regions [14]. The critical VL lesions included VLa, VApc, VLpv, and VLpd, which are densely connected with PFC [14]. The bottom row depicts high-resolution structural MRI of the VL patients acquired during the chronic stage following stroke. For patient VL1, we present the T2 brain scan rather than the T1 brain scan. Note that patient VL2 also had a small lesion in the left pallidum.
Details: VL patients' lesions involved VLa (green), VLpv (yellow), VLpd (violet), VApc (blue), VPla (light cyan), and VAmc (red). Note that only the VL3 lesion involved part of the anterior nuclei (AD and AV; dark cyan). The lesions in our MD patient mainly involved MDpc (green), CL (red), CM (blue), Pf (violet), VM (yellow), VPM (cyan), and VPLp (light green). The pulvinar patients' lesions involved PuA (green), PuL (violet), PuM (yellow), CM (red), MDpc (blue), and VPLp (cyan). The following abbreviations are used: VAmc, ventral anterior magnocellular; VApc, ventral anterior parvocellular; VLa, VL anterior; VLpd, VL posterior dorsal; VLpv, VL posterior ventral; VPla, ventral posterolateral anterior; MDpc, mediodorsal parvocellular; Pf, parafascicular; CL, central lateral; CM, central medial; VM, ventral medial; VPM, ventral posteromedial; PuM, medial pulvinar; PuL, lateral pulvinar; PuA, anterior pulvinar.

(see Figure 2E). Notably, memory of the cue was intact in the two VL patients (100% correct).

Experiment 3

In experiments 1 and 2, memory was assessed separately from the search task. It is possible that thalamic patients did not strongly commit the cue to WM despite it being search relevant and despite encouragement to use it strategically. Note, however, that this account would have predicted mere attenuation or absence of the WM bias rather than the reversed validity effect displayed by VL patients. Here, we included a memory test following the response to the search display in order to ensure that cues were in WM throughout the trials. Although delayed recognition memory was at ceiling (VL1 = 100% correct; VL2 = 97% correct), the same reversed validity effect was found (Figure 2F; see also Supplemental Experimental Procedures for a replication experiment).

This seemingly paradoxical effect is consonant with the view that thalamic insult triggers inhibition of any perceptual input that matches WM contents. We tested a crucial implication of this hypothesis in experiment 4.

Experiment 4

In experiments 1–3, WM contents were search relevant. However, recent research indicates that WM can automatically bias attention even when WM contents are irrelevant and detrimental in search [16, 17]: search is impaired when the WM content reappears as a search distracter as opposed to when it is absent [16]. This effect is contingent on participants holding the cue in WM because no attention bias is apparent when cues are merely attended (even in neurological populations [19, 20]; see [16, 17] for reviews).

To test the hypothesis that VL lesions result in the inhibition of memory-matching items, experiment 4 employed cues that were consistently invalid in search (Figure 2G). If the hypothesis was correct, then the patients would display better search performance than healthy controls in this invalid cueing protocol because the patients' attention would be repelled by rather than be attracted to the memory-matching distracters. We tested VL patients and healthy controls, along with patients Pulv2 and MD, who acted as a refined control for testing whether the VL patients specifically show a reversed invalidity effect.

We analyzed the cue-invalidity effects (invalid RT − neutral RT). Whereas healthy controls (n = 11) displayed attentional capture by irrelevant WM contents (i.e., slower search on invalid versus neutral trials), thalamic patients did not exhibit attentional capture (see Figures 2G and S2B for individual data).

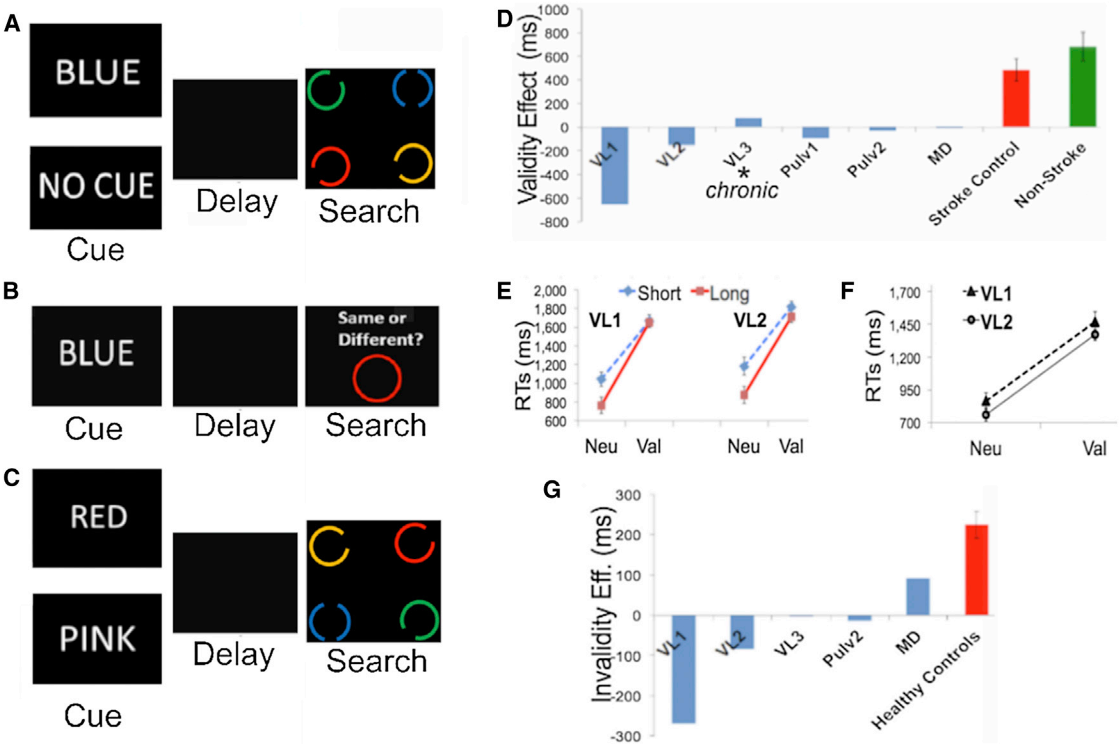

Figure 2. Patient Study: Trial Examples and Behavioral Data

(A) Memory-guided search task in experiments 1, 2, and 3. Participants were given either the name of a color cue, which always matched the search target (100% valid), or a neutral cue ("no cue"). This was followed by a search task that included finding the circle containing two gaps in the vertical plane and reporting whether it was located to the left or to the right of the central fixation. Participants responded via a button press.

(B) Delayed recognition task. In experiments 1 and 2, the ability to maintain the memory cue was assessed in a separate recognition task. Participants were required to remember the color word, and, following a 2 s delay, they were required to respond whether the colored circle matched or did not match the verbal cue. Experiments 3 and 4 incorporated a memory test after the search to ensure that the cue was held in memory across the delay.

(C) Invalid cueing in experiment 4. If the color word matched an item presented in the search display, this would never be the item surrounding the target (100% invalid trials).

(D) Experiment 1: Cue-validity effects on search (median neutral RT − median valid RT) for the thalamic patients and for the control groups (error bars show SEM of the cue-validity effect). The size of the cue-validity effects was reduced in the thalamic group relative to age-matched patients with lesions outside the thalamus ($n = 18$; $t(22) = -3.5$; $p = 0.002$; independent sample two-tailed t test) and in the nonstroke group of age-matched controls ($n = 22$; $t(26) = -3.4$; $p = 0.002$). Individual median RT data and SEM across the different validity conditions are presented in Table S2.

(E) Experiment 2: Search RTs as a function of cue-validity (Neu, neutral; Val, valid) and cue-search delay in patients VL1 and VL2. Note that performance of the patients in the short delay condition was compared with the performance of control groups from experiment 1, which had been tested with the same delay. Cueing effects differed significantly between the patients with lesions outside the thalamus and the two VL patients, and the same held for the comparison with the nonstroke group ($t > 11$, $p < 0.0001$).

(F) Experiment 3: Search RTs as a function of cue validity in the version of the task that incorporated a memory test after the search. VL patients showed reversed validity effects relative to the nonstroke controls and the stroke control group (all one sample $t > 10$, $p < 0.0001$).

(G) Experiment 4: Cue-invalidity effects (invalid RT − neutral RT) for the thalamic patients and the healthy controls. Importantly, we only analyzed search trials with correct recognition memory responses. Healthy controls ($n = 11$) displayed slower search in invalid trials relative to neutral trials ($F(2,10) = 50.47$; $p = 0.0001$). Invalidity effects were significantly lower in the thalamic patients than in the healthy controls ($t(26) = -3.38$; $p = 0.002$). This pattern of results held when the data were transformed to account for overall individual search latencies (Supplemental Experimental Procedures).

Most importantly, the two VL patients now displayed a reversed invalidity effect and responded faster in invalid trials compared to neutral trials (see Figure 2G). Notably, memory performance in these patients was high (VL1: 98% correct; VL2: 97% correct; VL3: 97.3% correct; Pulv2: 90% correct; MD: 85% correct).

Experiments 3 and 4 were conducted several months after stroke. We reiterate that patients VL1 and VL2 showed a similar reversed validity effect in the chronic and more acute stages, suggesting that the effect is unlikely to be the result of functional or maladaptive plasticity. Future research, however, ought to assess whether and how focal thalamic damage can trigger maladaptive functional reorganization changes in brain networks, which may further account for the impaired WM biasing of attention reported here.

fMRI Studies

Experiment 1: Anterior Thalamus Is Involved in WM Biasing

Given the limited sample size of our rare thalamic patients, we further probed the role of the VL and anterior thalamus in WM biasing of attention by using fMRI in healthy participants. Note that only one of the VL patients' lesions (i.e., VL3) comprised the more anterior thalamic nuclei (i.e., anterior dorsal [AD] and anterior ventral [AV]; Figure 1 and Table S1), described as part of a memory network including the hippocampus [15]. We propose that anterior nuclei, along with VL thalamus's contribution to action and oculomotor control [12, 13, 21, 22],

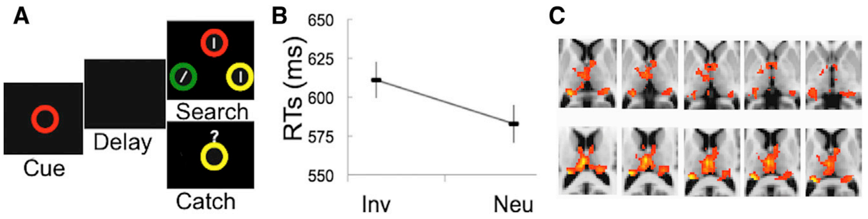

Figure 3. fMRI Experiment 1

(A) Task: A visual cue to be held in memory is presented. Following a delay, the search display appears. The task is to discriminate the orientation of the tilted bar (i.e., \ or /). An example of invalid trial is presented. Invalid trials were compared to a neutral baseline in which the cue was absent from search. In memory catch trials (20%), the search array was replaced by a recognition memory test.

(B) Search RTs across invalid and neutral trials (error bars show SEM). Search was impaired by the presence of an invalid WM distracter relative to the neutral baseline ($t(38) = 7.08$, $p < 0.00001$, two-tailed t test; Figure 3B), in keeping with an automatic bias of attention by WM contents. Memory accuracy was high (mean = 93% correct). Search accuracy was high (mean = 93.75% correct) and did not differ across the neutral and invalid trials ($t(38) = -0.842$; $p > 0.4$).

(C) Thalamic responses were enhanced by the reappearance of the cue relative to the neutral baseline.

may represent a nexus between mnemonic and attention control functions.

We tested this hypothesis further in an fMRI study of healthy volunteers (n = 39) by using a paradigm that assessed the automatic biases of attention through irrelevant WM content [23, 24] that was similar to experiment 4 except that (1) here, the cues were visual, (2) search displays were brief (0.1 s) to prevent saccades, and (3) the search target was a tilted line (/ or \) among vertical distracters (see Figure 3A and Supplemental Experimental Procedures).

Behavioral results were consistent with automatic WM biasing of search (Figure 3B). Given our a priori interest in the thalamus, neuroimaging analyses used masks of the right and left thalami. Relative to the neutral baseline, there were increased responses to the reappearance of a WM distracter in bilateral pulvinar thalamus (medial pulvinar [PuM], lateral pulvinar [PuL], and inferior pulvinar [PuI]), mediodorsal thalamus (mediodorsal parvocellular [MDpc]), and, more critically, (1) VL regions overlapping with the patients' lesion sites (ventrolateral anterior [VLa], ventrolateral posterior dorsal [VLpd], and ventral anterior parvocellular [VApc]) and (2) the more anterior thalamus bilaterally (including AV, anterior medial [AM], and AD) (see Figure 3C). These results survived correction for multiple comparisons within the thalamic regions of interest (ROIs) and across the whole brain. We also found activations in frontoparietal regions (Figure S3) and in the bilateral hippocampus (Montreal Neurological Institute [MNI]: 24, −20, −12 and −24, −26, −14). Given our a priori hypothesis concerning the thalamus, these activations are not discussed in great depth because they only provide correlational evidence, which, unlike our lesion evidence, precludes the formulation of causal inferences. We note that parietal and hippocampal responses have been recently associated with the strategic cognitive control over WM biases [25], and frontoparietal responses are classically involved in attention control [1].

Importantly, prior work has demonstrated that reappearance of WM contents is associated with increased neural responses relative to a nonrepetition baseline, whereas priming is associated with neural repetition suppression [26, 27]. Notably, we found no evidence for reduced responses to the reappearance of the memory cue in search. This is consistent with memory biases in this paradigm being contingent on WM [16, 17].

Experiment 2: Role of VL-Anterior Thalamus in Attention Biases Driven by Learning and Retrieval from Long-Term Memory

So far, the findings indicate a thalamic role in WM-based attentional control. However, it remains possible that the thalamus's role in attention may incorporate additional mnemonic processes, such as when information from long-term memory is brought "online" to guide behavior.

Here, we sought to further characterize the scope of memory types that may be involved in this thalamic control of attention. In the prior patient and fMRI study, the cueing of attention was accomplished via information in WM. We devised a new fMRI paradigm (n = 16) to assess whether the thalamus mediates attention guidance that relies on the learning of new stimulus-stimulus associations and their retrieval from long-term memory. Figures 4A and 4B illustrate the paradigm.

Behavioral results were consistent with the acquisition of knowledge about cue predictiveness as training developed and its use in driving attention (Figure 4C). fMRI analyses tested for linear learning trends associated with the predictiveness of the cues (predictive > nonpredictive) across training blocks and also tested for exponential trends because the behavioral manifestation of learning had an abrupt onset in block 4 (Figure 4C). Given that we were only interested in thalamic responses, the analyses were based on anatomical ROIs comprising the entire left and entire right thalami. Responses in anterior (AV), ventrolateral (VLpd, VApc), and mediodorsal (MDpc) regions of the right thalamus ($p < 0.05$, corrected for multiple comparisons; Figure 4D) were consistent with both linear and exponential learning trends in the learning protocol. No clusters survived this threshold in the left thalamus. No cortical responses survived whole-brain correction.

We then conducted additional unbiased ROI analyses based on the lesion evidence from our VL thalamus patients, and, accordingly, we used a 6-mm-radius spherical ROI that covered the anatomical lesion sites of our VL thalamus patients (centered at MNI −10, −10, 10, depicted in blue shading in Figure 4D). Voxels in left VApc and on the border of VLpd and MDpc thalamus tracked the predictiveness of the hiragana cues across training, consistent with an exponential learning trend ($p < 0.05$, voxel corrected for multiple comparisons across patient-based ROI; Figure 4D, voxels surrounded by white circles; these ROI results were corroborated by nonparametric permutation analyses).

Although there were significant responses in the left VL thalamus, it appears that right thalamic responses were more prominent in this learning protocol relative to fMRI experiment 1. It is possible that involvement of the right thalamus is stronger when learning of stimulus-stimulus associations needs to take place to guide attention. Future studies ought to assess this possibility.

Discussion

The present studies characterized the functional contribution of the VL and anterior regions of the human thalamus in the

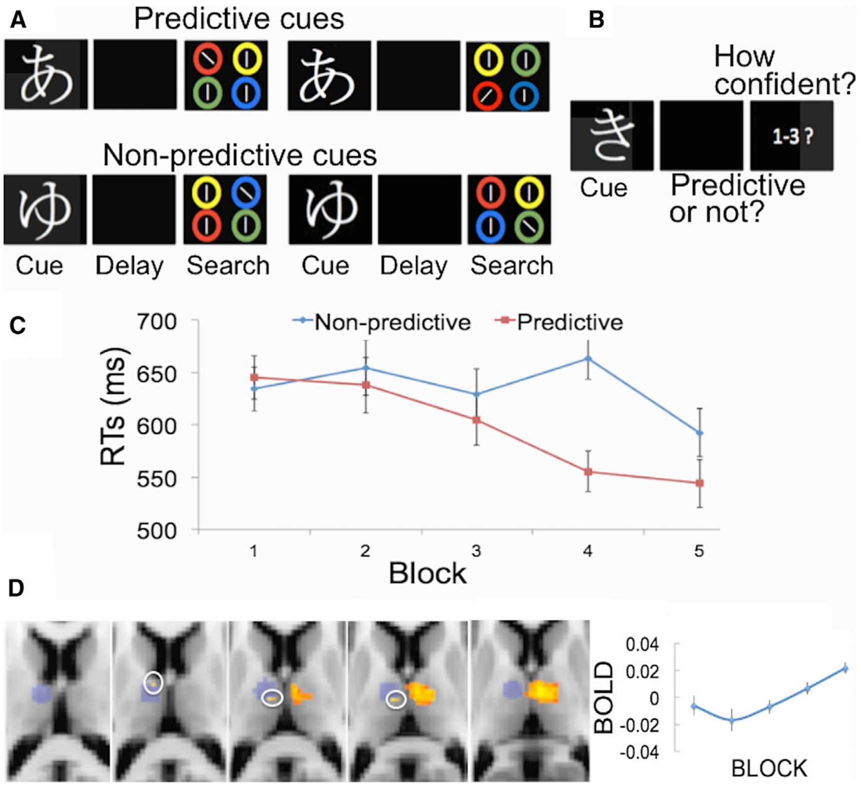

Figure 4. fMRI Experiment 2

(A) Learning and search phases. Participants were encouraged to form associations between the Japanese hiragana cues (not drawn to scale in the figure) and the colors of the circles surrounding the search target in order to boost search performance. The top row depicts a predictive trial (the hiragana cue is predictive of a red circle containing the tilted target). The bottom row depicts a nonpredictive trial (here, the hiragana cue is not associated with any target feature). Predictive and nonpredictive cues were presented randomly across trials. Four hiragana cues were 100% predictive (each of them was associated with a particular color surrounding the search target). Four different hiragana cues were neutral (not associated with any target features).

(B) Example of a recognition test trial. To further encourage learning, we presented recognition tests following each training block, which involved the presentation of hiragana probes, and participants were required to report whether or not the probes conferred predictive value for search and to rate how confident they were in their decisions on a confidence scale of 1–3.

(C) RTs for predictive cues showed evidence of learning across blocks compared to nonpredictive cues (error bars show SEM of the difference between predictive and nonpredictive RTs). Due to a technical issue, behavioral data during scanning could not be recorded for one participant. Data from the remaining 15 participants were entered into a 5 (block) × 2 (cue type: predictive and nonpredictive) repeated-measures ANOVA, which was performed over the median RTs of the correct search responses. There was a main effect of training ($F(4,56) = 5.01$; $p = 0.006$) such that search RTs became faster across the training blocks. Search performance was also faster following predictive rather than nonpredictive cues ($F(1,14) = 8.77$; $p = 0.01$). Importantly, these main effects were qualified by the presence of a significant interaction between cue type and block ($F(4,56) = 4.33$; $p = 0.007$). This interaction effect indicates that search performance became increasingly faster in predictive relative to nonpredictive trials as training developed. Search accuracy in the learning phase was very high (predictive trials = 92% correct; nonpredictive trials = 93% correct). There were no effects of block, cue, or interactions on search accuracy (for all: $p > 0.45$). Recognition data showed that learning of cue predictiveness improved with block ($F(4,56) = 6.5$; $p = 0.001$; Figure S4A; no other effects or interactions were evident; for all: $p > 0.3$). Likewise, memory confidence also increased with block ($F(4,56) = 9.4$; $p = 0.001$; Figure S4B). These results, along with the findings from the search latencies, are consistent with the acquisition of knowledge about cue predictiveness as training developed.

(D) Thalamus responses followed linear and exponential trends to the predictiveness of the cues during the learning. The graph displays the percent signal change of the difference between the parameter estimate for predictive minus nonpredictive trials from all significant voxels in the right thalamus ROI across blocks.

interplay between memory and attention. The lesion evidence demonstrates that thalamic lesions lead to impaired guidance of visual attention by WM contents. Remarkably, VL patients displayed reversed effects of cue validity independent of the relevance of the memory contents for search, even when they knew that cues were consistently associated with the target (experiments 1–3) or with a distracter (experiment 4). Importantly, the inability of the thalamic patients to use the cue in order to drive attention cannot be accounted for by an inability to retain the cue information in memory. These results are in keeping with the view that thalamic lesions disrupt the obligatory WM bias of attention that is observed in the healthy brain.

The VL thalamus, including VLa, VApc, and VLpd areas identified in our lesion and fMRI findings, is densely connected with cognitive control substrates in the prefrontal cortex (PFC) [14]. VL regions are also an integral part of the cortico-subcortical circuit comprising superior frontal regions and the superior colliculus for controlling eye movements [21, 22] and covert attention [28, 29]. This is one pathway through which the VL thalamus may be functionally relevant for the triggering of attention-biasing signals.

Furthermore, the anterior thalamus and the mamillothalamic tract are critical for normal memory function [30, 31], and the anterior nuclei also form part of a network comprising the hippocampus, mammillary bodies, and posterior cingulate cortex, which is relevant for recollective aspects of memory [15, 32]. Notably, recent research has demonstrated the role of the hippocampus in attentional guidance by WM and long-term memory [33, 34] as part of a network including the posterior cingulate and parietal cortex [34].

Based on the connectivity profile outlined above, it is possible that VL and anterior thalamus lesions lead to widespread damage of attention circuits—through connections with the superior frontal cortex and PFC [14]—and also memory circuits—via connections with hippocampus and posterior cingulate [15, 32]—which are key in controlling attention. Notably, whole-brain results from fMRI experiment 1 showed that the thalamus was coactivated along with frontoparietal areas and the hippocampus. Together, these findings help us understand the role of the thalamus in attention control by memory as part of a broader cortico-subcortical network. Thalamic damage may trigger disconnection between areas involved in perceptual selection and mnemonic control, leading to inhibition of memory-matching signals. Hence, the deployment of attention is directed away from those items.

These findings therefore indicate that in the normal functioning brain, the VL and anterior thalamus are key parts of the neural circuit mediating the automatic capture of attention by stimuli-matching WM contents, a notion that we confirmed in the first fMRI experiment.

Our lesion evidence enhances understanding of the nature of the functional role of the thalamus in memory and attention interactions beyond what could be anticipated from correlative fMRI findings alone. If the functional role of the thalamus was to regulate the activation state of memory representations based on their current relevance for task goals (e.g., downweighting representations associated with memory distracters for search), then we would have expected thalamic lesions to produce magnified attention biases by irrelevant contents held in memory (as previously found following PFC damage [20]). PFC lesions can lead to increased attentional capture by search distracters held in WM [20], suggesting that PFC mediates the capacity to shield irrelevant WM contents from the processes that guide search. Together, these findings indicate that the thalamus's role in WM biases of attention is dissociable from that of the PFC.

Memory biases of attention were attenuated in pulvinar patients. It has been debated whether the pulvinar's role in attention is related to the orienting or filtering of distracters [4, 9, 35]. Our lesion evidence is consistent with a pulvinar role in the orientation of attention, namely from the contents of WM. A filtering account would have predicted exacerbated distraction by irrelevant WM contents in pulvinar patients.

Finally, our second fMRI experiment showed that thalamic responses track the acquisition of stimulus-stimulus associations that are used to optimize attention in search. Hence, it demonstrates the flexible scope of memory types supported by the thalamus in the service of attention and how this can be shaped by experience and learning.

Animal studies point to a role of the anterior thalamus in memory and learning [36–39], and human studies implicated the VL thalamus in memory and language [40–42]. These findings, together with the present work, indicate that the anterior and VL thalamus can mediate attention control driven by information held in WM that is already consolidated in the cognitive repertoire (e.g., color cues) in addition to mediating the role of experience, the learning of new regularities, and the retrieval of learned information from long-term memory to guide attention.

Supplemental Information

Supplemental Information includes Supplemental Experimental Procedures, four figures, and four tables and can be found with this article online at http://dx.doi.org/10.1016/j.cub.2014.03.024.

Acknowledgments

This work was supported by grants from the MRC (UK, 89631) and the Bial Foundation. We thank Marty Sereno and the BUCNI staff for their kind support with the imaging protocol.

Received: February 1, 2014
Revised: February 24, 2014
Accepted: March 7, 2014
Published: April 17, 2014

References

1. Corbetta, M., and Shulman, G.L. (2002). Control of goal-directed and stimulus-driven attention in the brain. Nat. Rev. Neurosci. *3*, 201–215.
2. Heekeren, H.R., Marrett, S., and Ungerleider, L.G. (2008). The neural systems that mediate human perceptual decision making. Nat. Rev. Neurosci. *9*, 467–479.
3. Curtis, C.E., and D'Esposito, M. (2003). Persistent activity in the prefrontal cortex during working memory. Trends Cogn. Sci. *7*, 415–423.
4. Danziger, S., Ward, R., Owen, V., and Rafal, R. (2004). Contributions of the human pulvinar to linking vision and action. Cogn. Affect. Behav. Neurosci. *4*, 89–99.
5. Karnath, H.O., Himmelbach, M., and Rorden, C. (2002). The subcortical anatomy of human spatial neglect: putamen, caudate nucleus and pulvinar. Brain *125*, 350–360.
6. Saalmann, Y.B., Pinsk, M.A., Wang, L., Li, X., and Kastner, S. (2012). The pulvinar regulates information transmission between cortical areas based on attention demands. Science *337*, 753–756.
7. Shipp, S. (2004). The brain circuitry of attention. Trends Cogn. Sci. *8*, 223–230.
8. Shipp, S. (2003). The functional logic of cortico-pulvinar connections. Philos. Trans. R. Soc. Lond. B Biol. Sci. *358*, 1605–1624.
9. Strumpf, H., Mangun, G.R., Boehler, C.N., Stoppel, C., Schoenfeld, M.A., Heinze, H.J., and Hopf, J.M. (2013). The role of the pulvinar in distractor processing and visual search. Hum. Brain Mapp. *34*, 1115–1132.
10. Wurtz, R.H., McAlonan, K., Cavanaugh, J., and Berman, R.A. (2011). Thalamic pathways for active vision. Trends Cogn. Sci. *15*, 177–184.
11. Sommer, M.A., and Wurtz, R.H. (2004). What the brain stem tells the frontal cortex. I. Oculomotor signals sent from superior colliculus to frontal eye field via mediodorsal thalamus. J. Neurophysiol. *91*, 1381–1402.
12. Alexander, G.E., DeLong, M.R., and Strick, P.L. (1986). Parallel organization of functionally segregated circuits linking basal ganglia and cortex. Annu. Rev. Neurosci. *9*, 357–381.
13. Butler, E.G., Horne, M.K., and Rawson, J.A. (1992). Sensory characteristics of monkey thalamic and motor cortex neurones. J. Physiol. *445*, 1–24.
14. Behrens, T.E., Johansen-Berg, H., Woolrich, M.W., Smith, S.M., Wheeler-Kingshott, C.A., Boulby, P.A., Barker, G.J., Sillery, E.L., Sheehan, K., Ciccarelli, O., et al. (2003). Non-invasive mapping of connections between human thalamus and cortex using diffusion imaging. Nat. Neurosci. *6*, 750–757.
15. Aggleton, J.P., O'Mara, S.M., Vann, S.D., Wright, N.F., Tsanov, M., and Erichsen, J.T. (2010). Hippocampal-anterior thalamic pathways for memory: uncovering a network of direct and indirect actions. Eur. J. Neurosci. *31*, 2292–2307.
16. Soto, D., Hodsoll, J., Rotshtein, P., and Humphreys, G.W. (2008). Automatic guidance of attention from working memory. Trends Cogn. Sci. *12*, 342–348.
17. Olivers, C.N., Peters, J., Houtkamp, R., and Roelfsema, P.R. (2011). Different states in visual working memory: when it guides attention and when it does not. Trends Cogn. Sci. *15*, 327–334.
18. Krauth, A., Blanc, R., Poveda, A., Jeanmonod, D., Morel, A., and Székely, G. (2010). A mean three-dimensional atlas of the human thalamus: generation from multiple histological data. Neuroimage *49*, 2053–2062.
19. Soto, D., and Humphreys, G.W. (2006). Seeing the content of the mind: enhanced awareness through working memory in patients with visual extinction. Proc. Natl. Acad. Sci. USA *103*, 4789–4792.
20. Soto, D., Humphreys, G.W., and Heinke, D. (2006). Dividing the mind: the necessary role of the frontal lobes in separating memory from search. Neuropsychologia *44*, 1282–1289.
21. Tanaka, M., and Kunimatsu, J. (2011). Contribution of the central thalamus to the generation of volitional saccades. Eur. J. Neurosci. *33*, 2046–2057.
22. Kunimatsu, J., and Tanaka, M. (2010). Roles of the primate motor thalamus in the generation of antisaccades. J. Neurosci. *30*, 5108–5117.
23. Woodman, G.F., and Luck, S.J. (2007). Do the contents of visual working memory automatically influence attentional selection during visual search? J. Exp. Psychol. Hum. Percept. Perform. *33*, 363–377.
24. Soto, D., Heinke, D., Humphreys, G.W., and Blanco, M.J. (2005). Early, involuntary top-down guidance of attention from working memory. J. Exp. Psychol. Hum. Percept. Perform. *31*, 248–261.
25. Kiyonaga, A., Egner, T., and Soto, D. (2012). Cognitive control over working memory biases of selection. Psychon. Bull. Rev. *19*, 639–646.

26. Soto, D., Humphreys, G.W., and Rotshtein, P. (2007). Dissociating the neural mechanisms of memory-based guidance of visual selection. Proc. Natl. Acad. Sci. USA *104*, 17186–17191.
27. Miller, E.K., and Desimone, R. (1994). Parallel neuronal mechanisms for short-term memory. Science *263*, 520–522.
28. Cavanaugh, J., and Wurtz, R.H. (2004). Subcortical modulation of attention counters change blindness. J. Neurosci. *24*, 11236–11243.
29. Thompson, K.G., Biscoe, K.L., and Sato, T.R. (2005). Neuronal basis of covert spatial attention in the frontal eye field. J. Neurosci. *25*, 9479–9487.
30. Van der Werf, Y.D., Scheltens, P., Lindeboom, J., Witter, M.P., Uylings, H.B., and Jolles, J. (2003). Deficits of memory, executive functioning and attention following infarction in the thalamus; a study of 22 cases with localised lesions. Neuropsychologia *41*, 1330–1344.
31. von Cramon, D.Y., Hebel, N., and Schuri, U. (1985). A contribution to the anatomical basis of thalamic amnesia. Brain *108*, 993–1008.
32. Vann, S.D., and Albasser, M.M. (2009). Hippocampal, retrosplenial, and prefrontal hypoactivity in a model of diencephalic amnesia: Evidence towards an interdependent subcortical-cortical memory network. Hippocampus *19*, 1090–1102.
33. Stokes, M.G., Atherton, K., Patai, E.Z., and Nobre, A.C. (2012). Long-term memory prepares neural activity for perception. Proc. Natl. Acad. Sci. USA *109*, E360–E367.
34. Soto, D., Greene, C.M., Kiyonaga, A., Rosenthal, C.R., and Egner, T. (2012). A parieto-medial temporal pathway for the strategic control over working memory biases in human visual attention. J. Neurosci. *32*, 17563–17571.
35. Snow, J.C., Allen, H.A., Rafal, R.D., and Humphreys, G.W. (2009). Impaired attentional selection following lesions to human pulvinar: evidence for homology between human and monkey. Proc. Natl. Acad. Sci. USA *106*, 4054–4059.
36. Byatt, G., and Dalrymple-Alford, J.C. (1996). Both anteromedial and anteroventral thalamic lesions impair radial-maze learning in rats. Behav. Neurosci. *110*, 1335–1348.
37. Vann, S.D., and Aggleton, J.P. (2003). Evidence of a spatial encoding deficit in rats with lesions of the mammillary bodies or mammillothalamic tract. J. Neurosci. *23*, 3506–3514.
38. Aggleton, J.P., Neave, N., Nagle, S., and Hunt, P.R. (1995). A comparison of the effects of anterior thalamic, mamillary body and fornix lesions on reinforced spatial alternation. Behav. Brain Res. *68*, 91–101.
39. Parker, A., and Gaffan, D. (1997). The effect of anterior thalamic and cingulate cortex lesions on object-in-place memory in monkeys. Neuropsychologia *35*, 1093–1102.
40. Vilkki, J. (1978). Effects of thalamic lesions on complex perception and memory. Neuropsychologia *16*, 427–437.
41. Ojemann, G.A., Blick, K.I., and Ward, A.A., Jr. (1971). Improvement and disturbance of short-term verbal memory with human ventrolateral thalamic stimulation. Brain *94*, 225–240.
42. Ojemann, G.A. (1977). Asymmetric function of the thalamus in man. Ann. N Y Acad. Sci. *299*, 380–396.

Current Biology, Volume *24*

Supplemental Information

Thalamic Control of Human Attention

Driven by Memory and Learning

José de Bourbon-Teles, Paul Bentley, Saori Koshino, Kushal Shah, Agneish Dutta, Paresh Malhotra, Tobias Egner, Masud Husain, and David Soto

Supplemental Figures

Figure S1. Illustration of the lesion anatomy of the stroke patients with damage outside the thalamus, which is related to Figure 1.

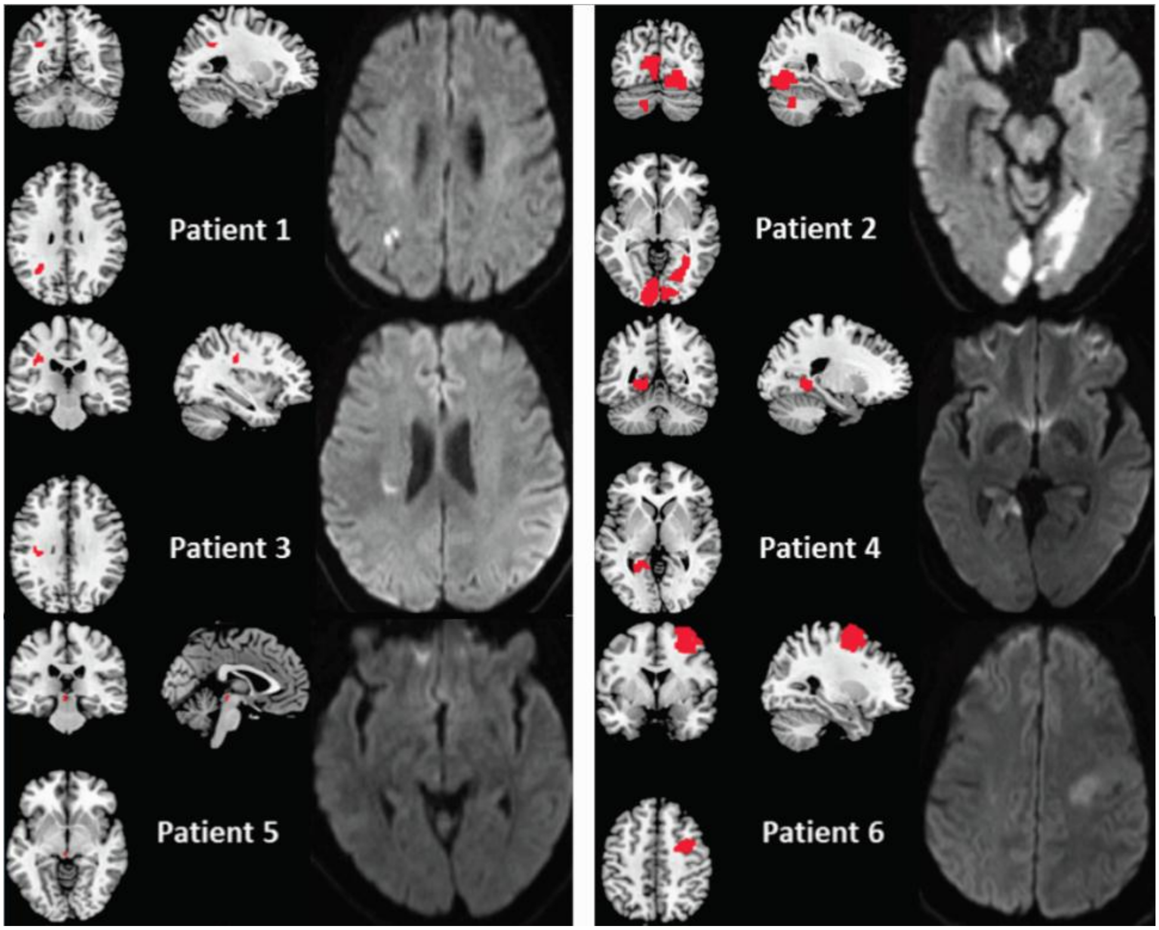

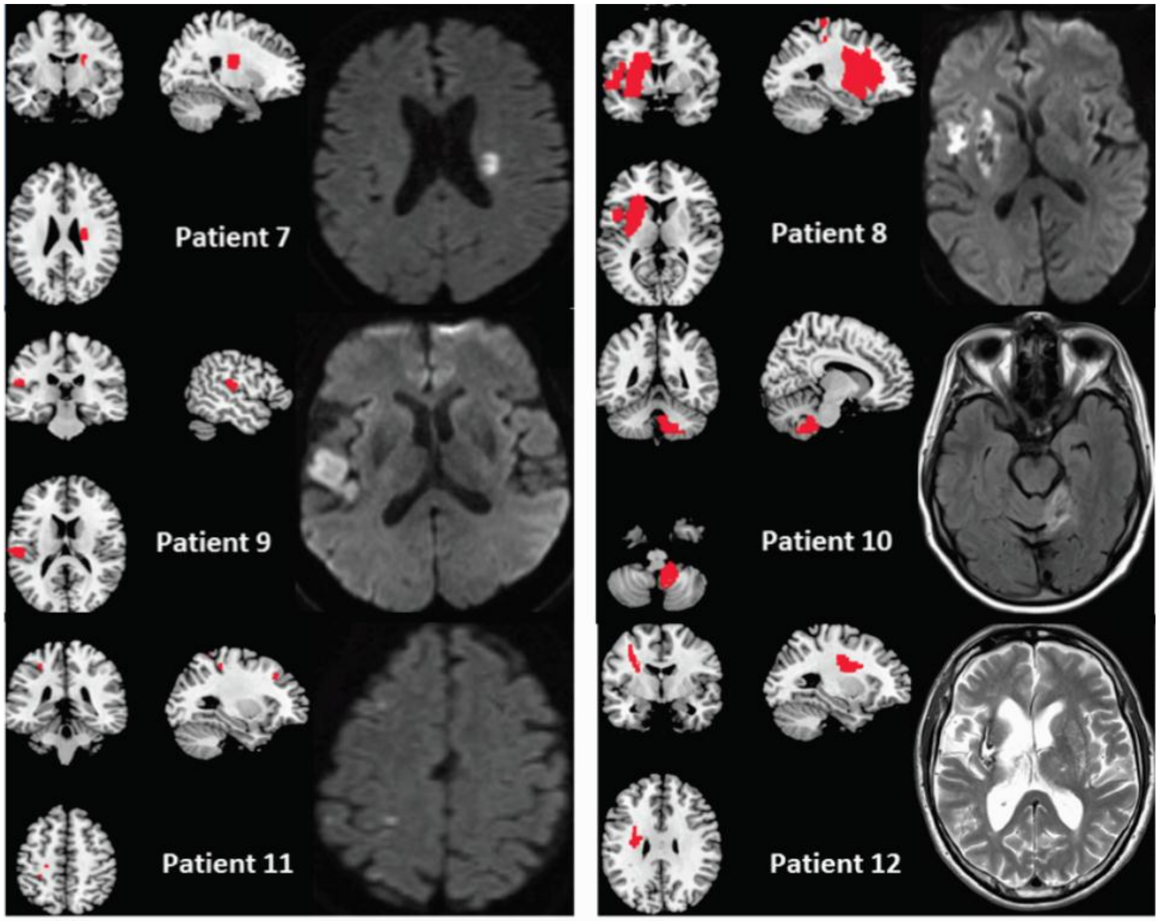
Patient 7
Patient 8
Patient 9
Patient 10
Patient 11
Patient 12

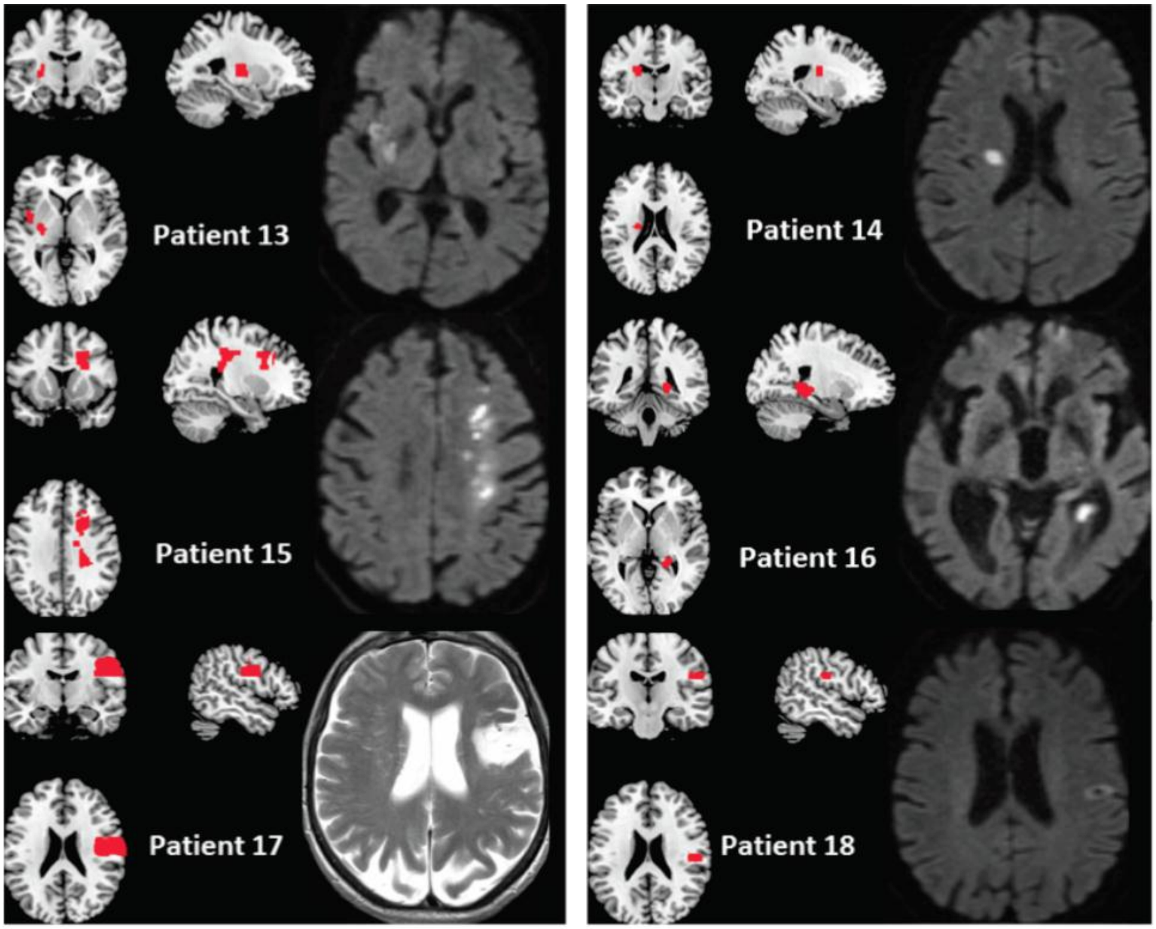

Patient 13
Patient 14
Patient 15
Patient 16
Patient 17
Patient 18

Figure S2. Behavioral data. (A) Median search reaction times in Experiment 1 as a function of cue validity (neutral in blue; valid in red) in the thalamic group, the stroke controls with lesions outside the thalamus and the non-stroke control group; this relates to Figure 2D. (B) Median Search RTs as a function of cue validity in Experiment 4 (invalid in blue, neutral in red) in the thalamic group and in the healthy age-match control group. This illustration relates to Figure 2G.

A

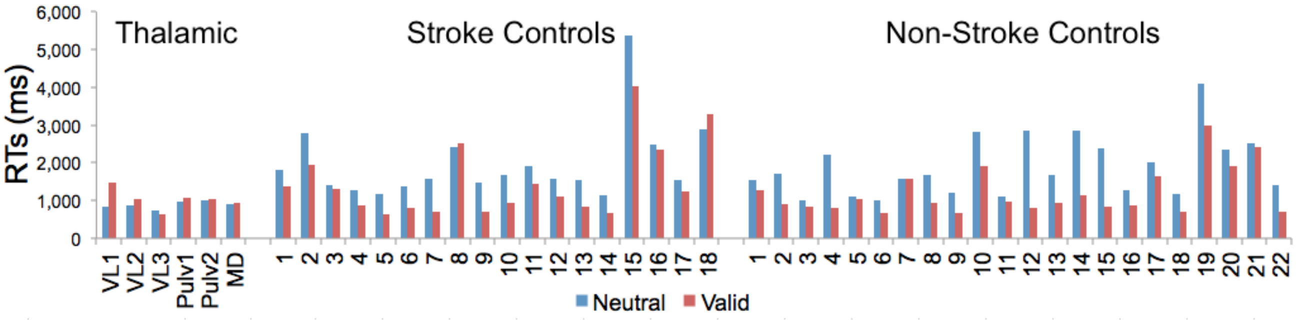

B

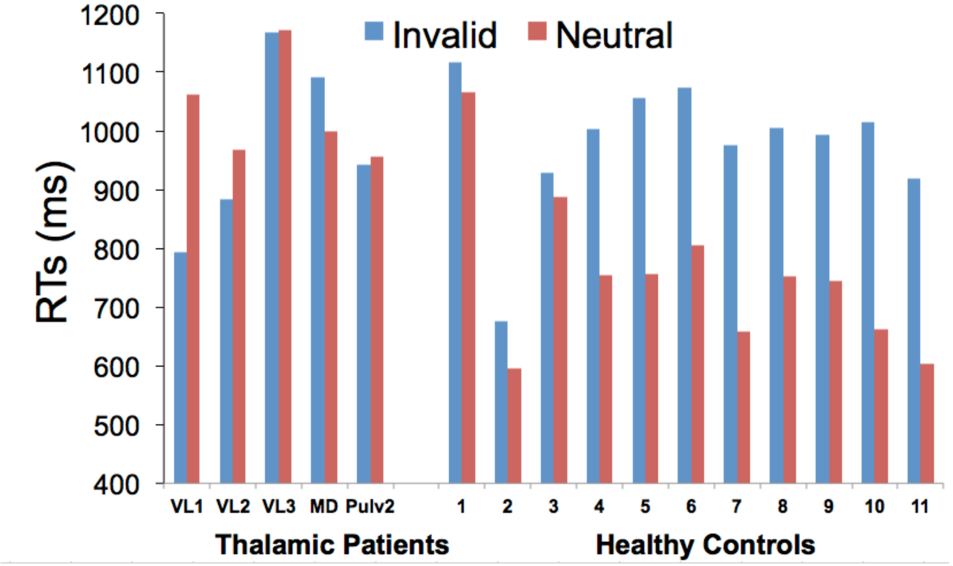

Figure S3. Reappearance effects in cortical responses, including posterior parietal cortex bilaterally, both superior (MNI: +/- 32 -54 50) and inferior parietal lobe bilaterally (MNI: +/-60 -30 30), and left rostral PFC (MNI: 20, 58, 0). This illustration is related to Figure 3C.

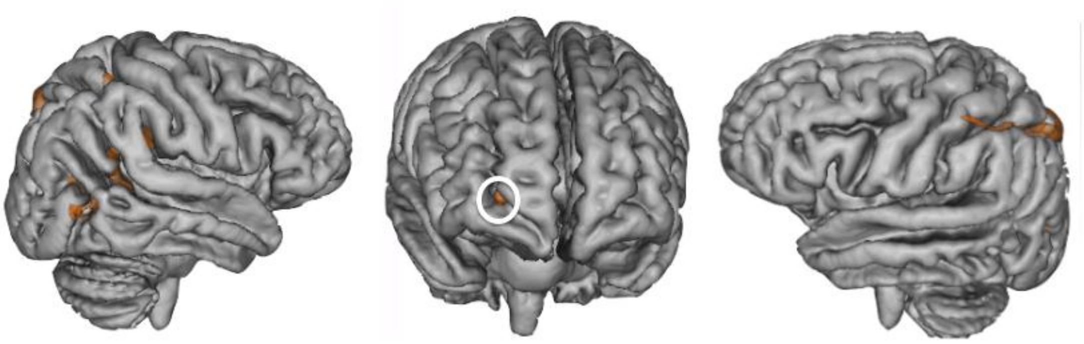

Figure S4. Recognition memory performance for the predictiveness of the hiragana cues (A) Memory accuracy (B) Confidence ratings (error bars= s.e.m.) related to Figure 4C.

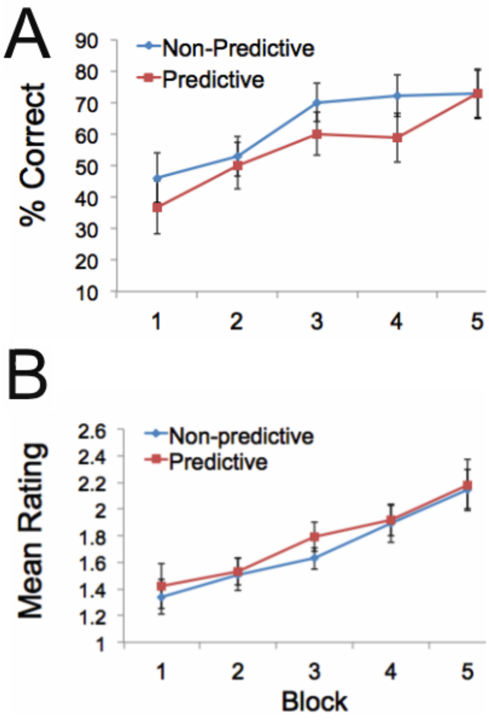

Supplemental Tables

Table S1. Percentage of thalamus nucleus occupied by the patient's lesion and cortical connectivity information. Thalamic nuclei are based on histology-based atlas [1].

Abbreviations:
Medial: MD: Mediodorsal nucleus | **MDmc:** Magnocellular part | **MDpc:** Parvocellular part | **CL**: Central lateral nucleus | **CeM:** Central medial nucleus | **CM:** Centromedian nucleus | **Pf:** Parafascicular nucleus

Posterior: **PuM:** Pulvinar Medial | **PuI:** Inferior | **PuL**: Lateral | **PuA**: Anterior | **LP**: Lateral posterior nucleus | **MGN**: Medial geniculate | **SG**: Suprageniculate nucleus | **Li**: Limitans nucleus

Lateral: **VPLa**: Ventral posterior lateral, anterior part | **VPLp**: posterior part | **VPM**: Ventral posterior medial nucleus | **VPI**: Ventral posterior inferior nucleus | **VLa**: Ventral lateral anterior nucleus | **VLpd:** Ventral lateral posterior dorsal | **VLpv:** posterior ventral | **VAmc**: Ventral anterior magnocellular | **VApc**: parvocellular part | **VM**: Ventral medial

Anterior: **AD:** Anterior dorsal | **AM:** Anterior medial | **AV**: Anterior ventral | **LD:** Lateral dorsal.

Cortical connectivity information
The superscripts indicate the cortical region that each damaged thalamic nuclei projects to according to the Oxford thalamic connectivity atlas derived from diffusion tensor imaging and probabilistic tractography [2].

temp: temporal | **pfc**: prefrontal | **ppc**: posterior parietal | **occ**: occipital | **premot**: premotor | **mot**: motor | **ss**: somatosensory

Patient

	VL1	VL2	VL3	MD	Pulv1	Pulv2
AD ^{temp}			44.4			
AM ^{temp}			1.0			
AV ^{temp}			92.5			
CL$^{\text{temp pfc}}$			48.0	63.8	71.4	3.5
CM ^{pfc}			39.3	1.0	73.4	55.8
CeM $^{\text{temp pfc}}$			94.2	7.5	9.8	
LD ^{temp}			53.7		29.9	
LP ^{ppc}		3.1	33.5	6.6	35.9	46.0
Li $^{\text{pfc, ppc}}$				68.3	68.3	6.2
MDmc $^{\text{temp pfc}}$			49.8	48.3	31.1	
MDpc ^{pfc}			66.0	78.8	75.4	3.3
MGN ^{ppc}					31.1	77.9
Pf ^{pfc}			27.9	89.6	41.5	21.9
Po $^{\text{ss ppc}}$				32.4	97.2	84.6

PuA $^{\text{ss mot ppc}}$		1.2	24.6	45.0	1.0	94.6
PuI $^{\text{ppc occ temp}}$					14.5	1.0
PuL ^{ppc}					78.8	91.8
PuM $^{\text{ppc occ temp pfc}}$				5.2	58.4	58.4
SG ^{ppc}				13.8	55.2	39.0
VAmc ^{pfc}		2.3	1.0			
VApc ^{pfc}	32.4	38.5	1.0			
VLa ^{pfc}	77.4	94.7	1.0	3.7	1.4	
VLpd ^{pfc}	3.8	33.9	1.0	5.2	32.9	
VLpv ^{pfc}	21.9	5.9	99.5	28.4	72.0	7.5
VM ^{pfc}	1.3	35.9	99.4	47.2	23.3	1.3
VPI ^{ss}		1.1	23.2		1.0	1.0
VPLa $^{\text{ss premot}}$	3.4	92.4	1.0		1.0	44.4
VPLp ^{ss}		29.5	8.7	14.6	96.0	56.6
VPM ^{pm}		8.6	61.8	6.2	86.2	76.3

Table S2. Median RTs and s.e.m across the different validity conditions in (A) Experiment 1 and (B) Experiment 4.

(A)

	Median RT		s.e.m	
	**Valid**	**Neutral**	**Valid**	**Neutral**
VL1	1482.00	833.50	81.54	90.33
VL2	1038.00	883.00	64.44	53.42
VL3	651.00	726.50	17.57	16.24
Pulv1	1069.00	971.00	51.57	46.35
Pulv2	1047.00	966.50	33.82	23.08
MD	922.00	917.00	33.98	27.96

(B)

	Median RT		s.e.m	
	**Invalid**	**Neutral**	**Invalid**	**Neutral**
VL1	792.50	1062.00	18.53	25.4
VL2	882.50	967.00	26.98	31.15
VL3	1168.00	1171.00	18.93	17.94
Pulv2	1091.00	999.00	26.48	22.06
MD	943.00	956.00	24.14	19.01

Table S3. Control group of patients with lesion outside the thalamus. Demographic description: sex/age, main lesion site. Most patients had ischemic stroke except patients 3 and 6 who were hemorraghic, and patient 8 who was both ischemic and hemorraghic.

Patients	Sex/Age	Main Lesion Site
1	Female/70	Left parietal
2	Male/74	Occipital and cerebellum
3	Male/58	Left white matter
4	Male/70	Left occipital lobe
5	Male/28	Midbrain
6	Male/66	Right superior frontal gyrus
7	Male/59	Right corona radiata
8	Male/59	Left insula, striatum and parietal
9	Female/66	Left temporoparietal
10	Male/55	Right cerebellum
11	Male/64	Left frontoparietal
12	Male/58	Left insula and right white matter
13	Male/74	Left insula, striatum
14	Male/51	Left striatum
15	Male/70	Right frontoparietal
16	Male/82	Right hippocampus
17	Male/68	Right pre/postcentral gyrus
18	Male/79	Right postcentral gyrus

Table S4. Performance of the thalamic patients in the Addenbrooke's Cognitive Examination (ACE) and Digit Span tests.

**Tests**	**Addenbrooke's Cognitive Examination (ACE)**						**Digit Span**	
Patients	Attention and Orientation	Memory	Fluency	Language	Visuo-spatial	Total ACE-R score	Forward	Backward
VL1	18/18	23/26	11/14	25/26	16/16	93/100	13/16	12/14
VL2	17/18	21/26	8/14	22/26	15/16	83/100	13/16	8/14
VL3	18/18	16/26	6/14	23/26	16/16	79/100	13/16	8/14
Pulv2	18/18	24/26	11/14	26/26	15/16	94/100	12/16	9/14
MD	18/18	22/26	9/14	24/26	11/16	84/100	11/16	4/14

Supplemental Experimental Procedures

Patient Studies

Participants

Six patients with thalamic lesions due to stroke, 18 patients with lesions outside the thalamus and 22 non-stroke patients were recruited at Charing Cross Hospital (London). The study was approved by the West London Research Ethics committee.

Patients VL1 (Male/74 years), VL2 (Female/59 years) and Pulv1 (Male/80 years) were initially tested in their acute stage in Experiment 1. Patient Pulv1 received no further testing whereas patients VL1 and VL2 received further testing up to 1 year following stroke. VL3 (Male/70 years) was recruited to take part in Experiment 1 and 4 five years after stroke and testing occurred in different sessions in separate days. Pulv2 (Male/68 years) and MD (Male/78 years) were tested in different sessions 3 and 10 months after stroke (in Experiment 1 and 4). All of them were right handed and suffered from ischemic stroke. Table S3 illustrates demographic and lesion site information for the control group of stroke patients and Fig. S1 depicts the anatomical site of their lesions.

The thalamic patients also completed the Addenbrooke's cognitive examination and standard forward and backward Digit Span tests test to gauge an indication of general cognitive ability (Table S4).

Task and Procedure

The tasks were programmed using E-Prime v2.0 (Psychology Software Tools Inc., Pittsburgh, USA; www.pstnet.com/eprime.cfm). Participants provided written informed consent to take part in the experiments. Prior to each task they received instructions on the validity of the cues for search and the relevant response mappings. Patients received practice trials until they felt comfortable performing the task.

Each search trial started with the presentation of a word (i.e. "Valid" or "Neutral") indicating whether or not the upcoming search trial would be validly cued (Experiment 1, 2, 3) to ensure the patients had foreknowledge of the cue validity prior to search. Valid and neutral trials were randomly presented. The experimenter then started the trial via

button press. A fixation point appeared for 1 s. Then the verbal cue (i.e. Red, Green, Blue, Yellow or Pink) appeared for 3 s. In the neutral condition the text “No cue” was displayed. Cue offset was followed by a 2 s delay (or 6 sec in Experiment 3) with a blank screen, which was then followed by the search display. There were four distinctive colored outline circles, each drawn any of five hues. Three of the circles had a gap either on the top-left or the bottom-right. The search target had two gaps, one on the top and on the bottom. The task was to locate the target circle (i.e. pressing one button for targets in the left side of the display and another button for right targets).

On valid trials (Experiments 1, 2 and 3), the verbal cue always specified the color of the search target and patients were instructed to keep that information online to maximize search performance. Because the word cues were consistently associated with the search target, they acted as ‘search template’, which according to extant theories of attention must be held in working memory (WM) to guide search [3]. Notably, given that the cue varied from trial to trial, there was indeed need for updating the content of the verbal cue in WM in order to drive attention. Further we elected to use color word cues in the Patient Study rather that colored shapes to avoid the presence of lower-level sensory priming effects. Finally, we note that our prior work in healthy subjects consistently indicates that verbal cues, like the ones used here, need in fact to be committed to WM in order to bias attention [4, 5].

On invalid trials (Experiment 4), the verbal cue never specified the color of the search target and rather the memory cue always matched a search distracter when reappeared in the search display. Participants received clear instructions about these contingencies and were encouraged to optimize search for the target circle with the 2 vertical gaps.

In addition to the search trials, Experiments 1 and 2 also included a recognition test in order to gauge the patient’s ability to keep the cues in memory. Note that the recognition test in Experiment 3 and 4 was embedded in the search trials and followed each search response while in Experiment 1 and 2 search and memory recognition testing were performed in separate blocks of trials (see below).

Each trial of the recognition test presented the observer with a color word for 3 s. Following a delay of 2 s with a blank screen, a recognition memory test appeared. Here a colored circle appeared and the task was to report whether or not the color word matched the circle's color. Patients gave their responses aloud, which were then recorded by the experimenter by pressing a different button for 'same' and 'different' responses.

The number of trials in Experiment 1 depended on patient's availability; patients VL1, VL2 and Pulv1 performed one block of 40 trials (20 valid and 20 neutral) except patients VL3, Pulv2 and MD who performed a total of 120 trials (60 valid and 60 neutral). Patients VL1, VL2, VL3 and Pulv1 performed the search task in Figure 2A and in addition performed 12 trials in the delayed memory test (Figure 2B). Patients Pulv2 and MD performed the same task but including a memory test for the initial cue at the end of each valid search trial. This was also due to time constrains in testing these patients. They were recruited in the chronic stage and we wished to ensure from the outset they attempted to commit the cue in WM throughout the trials. Patients VL1 and VL2 were subsequently tested in the same conditions and their data is presented as a control Experiment 3 to confirm that their unique performance impairment was not due to inability to remember the cue. Data from all the thalamic patients was presented together as Experiment 1 to illustrate the performance impairment of the thalamus group relative to the controls.

Experiment 2 involved the same task as Experiment 1 with the exception that the delay period between cue and search display was manipulated (i.e. short delay: 2 sec vs. long delay: 6 sec; randomly selected across trials and with equal probability of occurrence). Patients completed 128 trials with 32 trials per each validity and delay condition. Each patient also performed the 12 trials in the delayed memory task.

Experiment 3 was similar to Experiment 1 except that the recognition test was embedded in the search trials and therefore memory probes followed each search response. There were 60 trials (30 valid and 30 neutral trials; randomly selected.

In Experiment 4, the memory cue was always invalid when it reappeared in the search display. There were 120 trials (60 invalid and 60 neutral).

fMRI Studies

Experiment 1

Participants

Thirty-nine participants took part (20 females; age range: 21-23 years) in return for £20. They were native-English speakers and naive with regard to the experimental aims and hypothesis. No participant had a prior history of neurological or neuropsychiatric disorders, and all had normal vision. This fMRI experiment is part of a bigger study for which DTI and resting state BOLD data were collected and that will be reported in a separate paper.

Task and Procedure

Each trial began with a fixation display for 0.5 sec that was followed by 1 s delay before the onset of the memory cue. The cue appeared for 0.25 s and was a colored circle drawn in one of four different colors. The offset of the cue was followed by a delay period of 1 s. Then, the search display was presented for 0.1 s and was composed of three colored circles at the corners of an imaginary triangle. Each circle contained a line; two of the lines were vertical while the target line was tilted 16° either to the left or right. The participants' task was to indicate the orientation of the tilted line via button press during a time window of 1 s. Target location and orientation were randomly selected. The memory and search circles were randomly chosen from one of four colors (red, blue, green, or yellow). Each circle was a color singleton in the search display. The cues could be ’invalid’ or ‘neutral’ depending on whether they surrounded a search distracter or did not reappear in the search display. To ensure that any WM effect on search occurred automatically, namely, without intention on the part of the observer (cf. obligatory coupling between memory and attention) it is fundamental to avoid the contribution of top-down strategic factors (i.e. expectancy that the contents of WM may be associated with the search target) [6, 7]. Accordingly, we made sure participants were fully aware of the invalidity of the memory cues for search by blocking the invalid and neutral conditions. There were 6 invalid and 6 neutral blocks (Inv-Neu-Inv-Neu-Inv-Neu-Inv-Neu-Inv-Neu-Inv-Neu) with each including 10 trials (8 search and 2 memory catch trials, see next). We ensured that participants held the cues in WM throughout the trials by using memory “catch” trials (20%) where a memory probe was presented for 1.5 s instead of the visual search display. The probe consisted of a colored circle, and

participants had to provide a match/mismatch response, indicating whether or not the probe was the same color as the memory cue.

Participants received a 5 s instruction display at the beginning of each block, which informed about the nature of cue in the subsequent trials. Participants received clear instructions about the presence of memory “catch” trials. Participants completed several practice sessions outside the scanner, initially with a visual search display duration of 0.5 s followed by a search display duration of 0.1 s until performance was above 90% correct. They were told to avoid eye movements during the search, and we used the very brief search display time (0.1 s) to discourage eye movements.

Scanning Parameters

We used a Siemens Avanto 1.5T MRI scanner and a 32-channel head coil. Sagittal T2-weighted volumes were acquired (220 volumes) with a field of view (FOV) of 205 × 205 mm, repetition time (TR) of 2.5 s, echo time (TE) of 44 ms, and slice thickness of 3.2 mm. A six minutes T1-weighted structural scan was also collected.

Data Analysis

We used FEAT (fMRI Expert Analysis Tool) Version 6.0, as part of FSL (www.fmrib.ox.ac.uk/fsl). The first four EPI volumes were removed to account for T1 equilibrium effects. Non-brain removal was performed using Brain Extraction Tool [8]. Motion correction of functional scans was carried out using FMRIB’s Linear Image Registration Tool [9]. We applied a 100 s high-pass temporal filtering to remove low frequency noise, and spatial smoothing using a FWHM Gaussian kernel of 6 mm. Time-series statistical analyses were conducted using FILM (FMRIB’s Improved Linear Model) with local autocorrelation correction [10]. The data were analyzed using voxelwise time series analysis within the framework of the general linear model. A design matrix was generated with a double-gamma hemodynamic response function and its first temporal derivative; invalid and neutral blocks were modelled as regressors of interest. The 5 s instruction prior to each block and the presence of catch trials and error responses were modelled as regressors of no interest. We obtained contrasts of parameter estimates for invalid > (<) neutral for each individual, which were carried forward to a higher-level analysis using FLAME 1+2 (FMRIB’s Local Analysis of Mixed Effects) in the form of one-sample t-test to assess consistent regions of activation across participants. The

data were registered to individual high-resolution structural images using FLIRT Boundary-Based Registration [11], and then co-registered into standard MNI space. We report thalamic maps of BOLD responses thresholded using clusters determined by a voxelwise threshold of $Z = 2.3 (P<0.01)$ and a cluster significance threshold of $P=0.05$, corrected for multiple comparisons (using Gaussian Random Field Theory) within the left and right thalamus ROIs [12]. For the more exploratory analyses of cortical activations we followed Hayasaka and Nichols [13] and employed a more stringent voxelwise cluster-forming threshold of $Z>3.09$ ($P<0.001$) along with a cluster significance threshold of $P=0.05$, corrected for multiple comparisons across the whole brain.

Experiment 2

Participants

Sixteen new right-handed healthy participants (seven females; age range: 18-31 years) participated in the study in return for £20. Due to a technical problem behavioural data from one participant could not be recorded.

Task and Procedure

Learning study phase

Each trial of the learning phase began with the presentation of one of eight possible Japanese hiragana symbols (the cues) for 0.5 s. Then, a blank screen was presented for 1.5 s, which was followed by a search display comprising four outline circles in red, green, yellow and blue displayed at the corners of an imaginary square. Three of the colored circles contained a vertical line and the remaining circle contained a tilted line (the search target). The search task was the same as in fMRI Experiment 1. Search display appeared for 0.1 s to discourage eye movements. Four of the hiragana cues were predictive of the color of the circle surrounding the search target; each of the 4 predictive hiragana cues was associated with one of the four colors. The remaining 4 hiragana cues were non-predictive and not associated with any target feature. The color of the circle surrounding the search target was randomly selected on these non-predictive trials. The inter-trial time interval (ITI) was jittered between 2.5 and 4 s, with a pseudo-exponential distribution (50% of ITIs were 2.5 s, 25% of ITIs were 3 s, 12.5% were 3.5 s, and 12.5% were 4 s), to facilitate the independent estimation of BOLD responses across trials. A central fixation point was presented during the ITI. The task

was composed of 5 ‘search’ blocks of 16 trials each (8 predictive and 8 non-predictive; each symbol was displayed 2 times per block randomly across trials).

Each ‘search’ block was followed by a recognition test to ensure that participants were indeed attempting to learn the predictive value associated with the different hiragana cues. Each recognition block began with a 2.5 s instruction display. On each trial, participants were presented with one of the hiragana cues for 0.5 s, followed by a blank screen for 2.5 s during which participants had to indicate via button press whether the symbol was predictive or non-predictive. Following this, participants were asked to rate how confident they were of their decision during a time window of 1.5 s. A 1-3 rating scale was used (1: no confidence; 2: medium confidence; 3: high confidence). Each recognition block comprised 8 trials, one for each of the hiragana cues used (4 predictive and 4 non-predictive).

Prior to scanning, participants received instructions and training on the task. Note that the hiragana cues used in the training phase were different from the ones used in the scanner. Participants were explicitly trained to maintain central fixation throughout the trials and to avoid eye movements like in fMRI Experiment 1.

Scanning Parameters

These were identical to fMRI Experiment 1 except that 342 volumes were acquired here. The duration of the scan was based on pilot testing which determined the amount of exposure needed to observe learning effects.

Data Analysis

The analysis pipeline was identical to Experiment 1 unless otherwise noted. The trials of the learning protocol were modelled from the onset of the hiragana symbols with duration of 2.1 s (including 0.5 s cue exposure, 1.5 s blank screen delay and 0.1 s search display). We separately modelled the predictive and non-predictive cues for each of the five learning blocks independently. The recognition trials were modelled from the onset of the memory probes (predictive and non-predictive) for each of the five recognition blocks separately. We also included regressors for the onset of the confidence rating periods. Finally we modelled the search error trials and the instruction display periods preceding each of the search and recognition test phases.

We obtained contrasts of parameter estimates for predictive > (<) non-predictive trials, which were carried forward to second-level within-subject analysis (fixed effects) to test for linear and exponential trends across the 5 blocks. Finally, we performed group-level analyses as in fMRI Experiment 1. Co-registration of the functional data to standard space and the statistical thresholding of the functional activations in the thalamus ROI analyses were also performed as in Experiment 1. Additional unbiased ROI analyses based on the area of overlap of the VL patient lesions were also performed using a 6 mm radius sphere; we employed parametric tests and voxelwise correction for multiple comparisons ($P<0.05$) using GRF-theory-based maximum height threshold, and also nonparametric permutation testing to further corroborate the results. Five thousand random permutations were calculated to create the null distribution for assessing the test statistics.

Supplemental Analyses

Patient Study

Experiment 1

To confirm that the results held when the data were transformed to account for variation in RT across individuals, we computed the individual cuing effect as (Neutral RT – Valid RT)/(Neutral RT - Valid RT). Analyses confirmed that thalamic patients had lower cuing effect relative to patients with lesions outside the thalamus ($t(22)=-4.01$, $p=0.001$) and the non-stroke group ($t(26)=-4.41$, $p=0.0001$), which did not differ ($p>0.343$).

Experiment 4

We computed the individual cue invalidity effect as (Invalid RT - Neutral RT)/(Invalid RT - Neutral RT) and confirmed the size of the invalidity effect in the thalamus patients was significantly lower than in the healthy controls ($t(14)=-4.374$, $p=0.0001$).

Supplemental Data

Replication of the 'reversed' validity effect

Patients VL1 and VL2 took part in this replication experiment conducted one month later in which we also varied the delay between memory and search. There were 64 trials (32 valid and 32 neutral). The same reversed validity effect was evident, with faster search RTs in neutral relative to valid conditions. Mean search accuracy was high (VL1: Neutral: 97%, Valid: 81%; VL2: Neutral: 100%, Valid: 100%).

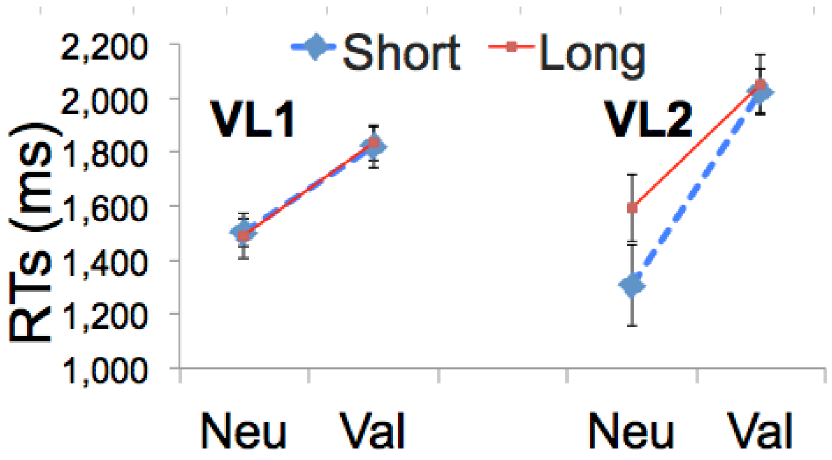

Supplemental References

S1. Krauth, A., Blanc, R., Poveda, A., Jeanmonod, D., Morel, A., and Szekely, G. (2010). A mean three-dimensional atlas of the human thalamus: generation from multiple histological data. Neuroimage *49*, 2053-2062.

S2. Behrens, T.E., Johansen-Berg, H., Woolrich, M.W., Smith, S.M., Wheeler-Kingshott, C.A., Boulby, P.A., Barker, G.J., Sillery, E.L., Sheehan, K., Ciccarelli, O., et al. (2003). Non-invasive mapping of connections between human thalamus and cortex using diffusion imaging. Nat Neurosci *6*, 750-757.

S3. Olivers, C.N., Peters, J., Houtkamp, R., and Roelfsema, P.R. (2011). Different states in visual working memory: when it guides attention and when it does not. Trends Cogn Sci *15*, 327-334.

S4. Soto, D., and Humphreys, G.W. (2008). Stressing the mind: the effect of cognitive load and articulatory suppression on attentional guidance from working memory. Percept Psychophys *70*, 924-934.

S5. Soto, D., and Humphreys, G.W. (2007). Automatic guidance of visual attention from verbal working memory. J Exp Psychol Hum Percept Perform *33*, 730-737.

S6. Woodman, G.F., and Luck, S.J. (2007). Do the contents of visual working memory automatically influence attentional selection during visual search? J Exp Psychol Hum Percept Perform *33*, 363-377.
S7. Soto, D., Heinke, D., Humphreys, G.W., and Blanco, M.J. (2005). Early, involuntary top-down guidance of attention from working memory. J Exp Psychol Hum Percept Perform *31*, 248-261.
S8. Smith, S.M. (2002). Fast robust automated brain extraction. Hum Brain Mapp *17*, 143-155.
S9. Jenkinson, M., Bannister, P., Brady, M., and Smith, S. (2002). Improved optimization for the robust and accurate linear registration and motion correction of brain images. Neuroimage *17*, 825-841.
S10. Woolrich, M.W., Ripley, B.D., Brady, M., and Smith, S.M. (2001). Temporal autocorrelation in univariate linear modeling of FMRI data. Neuroimage *14*, 1370-1386.
S11. Greve, D.N., and Fischl, B. (2009). Accurate and robust brain image alignment using boundary-based registration. Neuroimage *48*, 63-72.
S12. Worsley, K.J. (2001). Functional MRI: An Introduction to Methods, (Oxford University Press.).
S13. Hayasaka, S., and Nichols, T.E. (2003). Validating cluster size inference: random field and permutation methods. Neuroimage *20*, 2343-2356.
